# A sex- and gender-based analysis plus of frequent healthcare utilization among individuals living with chronic pain: a cohort study

**DOI:** 10.1186/s12913-025-13374-5

**Published:** 2025-10-01

**Authors:** Marimée Godbout-Parent, Nancy Julien, Hermine Lore Nguena Nguefack, M. Gabrielle Pagé, Line Guénette, Lucie Blais, Anaïs Lacasse

**Affiliations:** 1 Département des sciences de la santé, Université du Québec en Abitibi-Témiscamingue (UQAT), Rouyn-Noranda, Québec Canada; 2 Centre de recherche du Centre hospitalier de l’Université de Montréal (CRCHUM), Montréal, Québec Canada; 3 Département d’anesthésiologie et de médecine de la douleur, Faculté de médecine, Université de Montréal, Montréal, Québec Canada; 4 Faculté de pharmacie, Université Laval, Québec, Québec Canada; 5 Axe Santé des populations et pratiques optimales en santé, Centre de recherche du CHU de Québec, Université Laval, Québec, Québec Canada; 6 Faculté de pharmacie, Université de Montréal, Montréal, Québec Canada

**Keywords:** Chronic pain, Sex, Gender, Social determinants of health, Frequent users, Medical visits, Healthcare utilization, Sex- and gender-based analysis plus, SBGA+, Cluster analysis, Cohort study

## Abstract

**Background:**

Chronic pain (CP) affects up to 1 in 4 individuals and disproportionately impacts women, gender minorities, and other equity-deserving groups, highlighting the need for an equity-oriented approach to provide optimal care. The relationship between sex, gender, and frequent healthcare utilization remains underexplored among individuals living with CP. Therefore, this study aimed to examine the associations between sex, gender and frequent medical care utilization among persons living with CP.

**Methods:**

The COPE Cohort, composed of persons living with CP, was formed by linking a web-based questionnaire with health administrative databases. Frequent medical care users were defined as the top 10% of our sample with the highest number of all-cause medical visits in the year following the completion of the questionnaire (including outpatient and emergency department visits). Sex (male / female), gender identity (men / women / gender-diverse), and gender-stereotyped personality traits (masculine / feminine / androgynous / undifferentiated; Bem Sex-Role Inventory) were analyzed. Cluster analysis was used to create intersecting sociodemographic subgroups (incorporating sex, gender, region of residence, country of birth, education level, employment status, and age).

**Results:**

Among 895 participants, 95 were classified as frequent medical care users (10% cut-off: ≥13 visits/year). The proportion of frequent users varied across sex (male: 3.9% vs. female: 12.0%, *p* = 0.003) and gender identity (men: 4.0% vs. women: 12.1% vs. gender-diverse: 0%, *p* = 0.009), but not by gender-stereotyped personality traits subgroups. Multivariable logistic regression showed that the subgroup labelled ‘unemployed more educated women’ (compared to ‘men’) had increased odds of being frequent medical care users (OR: 3.93, 95%CI: 1.44–10.80).

**Conclusion:**

Based on our results, we emphasize the need for clinicians and decision-makers to adopt an integrated approach that considers not only clinical, but importantly, socioeconomic profiles for effective healthcare.

**Supplementary Information:**

The online version contains supplementary material available at 10.1186/s12913-025-13374-5.

## Introduction

Chronic pain (CP), defined as pain that persists or recurs beyond three months, affects up to 1 in 4 individuals [1, 2]. Research shows that CP disproportionately impacts women, sexual and gender minorities, and other equity-deserving groups, highlighting the need for an equity-oriented approach to care [3]. International and national organizations, along with several authors emphasize the urgent need for more equitable and effective treatments for everyone living with CP [3–7].

Equitable and tailored treatments require consideration of biological, psychological, social, and cultural factors [8], necessitating a multidisciplinary and multimodal approach for effective pain management [9, 10]. CP management, however, remains suboptimal due to barriers such as limited access to healthcare professionals adequately trained in pain management, clinician biases, unclear care pathways, and insufficient community resources [11]. These challenges are exacerbated by socioeconomic and sociocultural factors [4], with equity-deserving groups facing additional difficulties, such as the minimization of women’s health concerns [12], undertreatment of pain for racialized individuals [13], and limited access to analgesics in low-income countries [14]. In addition to numerous treatment barriers, we must consider complex care management and healthcare systems that are difficult to navigate. The absence of clear care pathways leads patients to consult multiple healthcare professionals in a fragmented manner [15].

Notably, the phenomenon of frequent healthcare users, a small group of patients accounting for a high number of healthcare visits [16], highlights the need to identify their characteristics and unmet needs. While some chronic conditions may require frequent visits for appropriate care, frequent healthcare utilization is mainly associated with negative factors such as mental health issues, substance abuse, chronic diseases, insurance challenges, and lower employment rates [17]. Frequent healthcare utilization contributes significantly to healthcare costs [18] and care is often deemed inappropriate [19]. Persons living with CP (compared to the general population) are more likely to visit their physician, the emergency department, other healthcare professionals [20], and to be frequent healthcare users [21], especially when pain severely impacts daily activities and causes high disability [21].

**Fig. 1:**
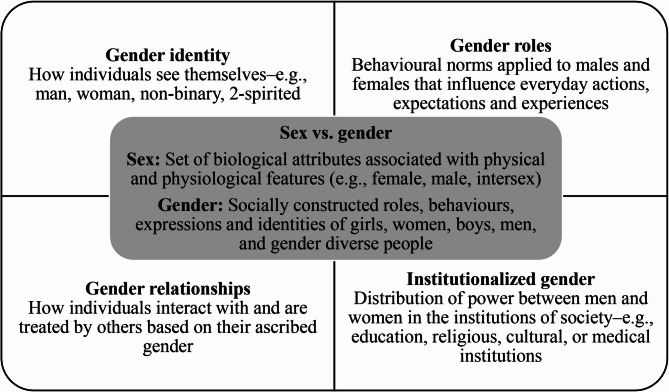
Definitions of sex, gender and examples of gender constructs [27, 28]

The relationship between sex, gender (Fig. 1), and frequent healthcare utilization remains underexplored among individuals living with CP. While some studies focus on emergency department visits [17, 22, 23] or primary care settings [17, 24], they often overlook sex and gender as key determinants. Gender, specifically, has not been considered in studies examining frequent healthcare utilization among CP patients. Moreover, although recommended in the Canadian Institutes of Health Research’s Institute of Gender and Health’s new action plan [5], very few studies in the health field have gone beyond describing sex and gender differences to examine and understand the underlying factors, processes, and their intersections. Centering sex and gender while considering their intersections with other identities (e.g., age, education, race) is essential for a comprehensive understanding of health [5, 25, 26]—particularly in the context of frequent healthcare utilization. Therefore, this study aimed to examine the associations between sex, gender, and frequent medical care utilization among persons living with CP, while incorporating other social determinants of health such as the region of residence, country of birth, education level, employment status, and age.

## Methods

This study is reported in accordance with the Sex and Gender Equity in Research (SAGER) guidelines checklist [29, 30].

### Study population

This research utilized data from the ChrOnic Pain trEatment (COPE) Cohort [31], which aims to understand treatment utilization among persons living with CP. The dataset is derived from a combination of two data sources: (1) a cross-sectional web-based questionnaire conducted between June and October 2019, involving 1,935 participants living with CP, and (2) longitudinal health administrative databases from the province of Quebec (Canada) that include universal public health insurance data. To be eligible, participants had to live with CP (pain that persists or recurs for more than three months [32]), be at Least 18 years old, reside in Quebec, and be able to complete a web-based questionnaire in French. In the province of Quebec, 93.7% of the population reports being able to speak French well enough to hold a conversation in the language [33]. This study involved COPE Cohort participants who provided their health insurance number and consented electronically to data linkage, with successful matching performed by the *Institut de la Statistique du Québec* (ISQ) (*n* = 895). No selection criteria based on prior healthcare utilization were applied, which allowed us to work with a community-based sample. Participants in the COPE cohort (and those who consented to data linkage which formed the present study sample) were deemed comparable to random (representative) samples of Canadians living with CP in terms of age, employment status, education level, pain duration, pain intensity, and pain location [31]. However, women were overrepresented in our sample (84% vs. 55–65% in random samples of persons living with CP), highlighting the importance of stratifying results by sex and gender in studies conducted with COPE cohort data to assess the impact of this overestimation and adjust accordingly. This overrepresentation is likely due to the web-based recruitment strategies and questionnaire administration we employed, known to reach more women. In fact, women tend to use Facebook [34] and to work in online environments more than men [35]. This further underscore the importance of stratifying analyses by sex and gender, which is the primary aim of our study.

To better contextualize the study to an international readership, it is relevant to clarify the understanding of gender (socially prescribed and experienced dimensions of “femaleness” or “maleness” in a society [36]) within our study population. The province of Quebec is characterized by its unique cultural and linguistic context as profound changes have taken place over the past four decades [37, 38] for gender identities (how individuals perceive themselves [27]) and gender roles (behavioural norms applied to males and females in societies that influence their everyday actions, expectations, and experiences [27]). The affirmation of diverse gender identities is increasingly valued [39]. Also, broader societal changes have empowered women and recognized men’s involvement in traditionally feminine roles, such as childcare and housework [40].

### Data sources

The content of the COPE web-based questionnaire was informed by established domains and measures in the field of CP, including the Methods, Measurement and Pain Assessment in Clinical Trials (IMMPACT) [41], the Canadian Minimum Dataset for chronic low back pain research [42], and the Quebec Pain Registry [43]. The questionnaire also integrated critical indicators recommended by the Canadian Registry Working Group of the Strategy for Patient-Oriented Research (SPOR) Chronic Pain Network (CPN) [44, 45]. The complete questionnaire is available in Supplementary content 1 (Original French version and English translation for publication purposes). As for health administrative databases, the entire population is covered by a provincial universal health insurance program administered by the *Régie de l’assurance maladie du Québec* (RAMQ) [46]. The health insurance covers the cost of outpatient medical visits, emergency department visits, hospitalizations, and procedures offered to all residents (8.5 million people) [47].

The project was approved by relevant authorities: (1) *Université du Québec en Abitibi-Témiscamingue* (#2018-05-Lacasse, A.), (2) *Commission d’accès à l’information du Québec* (#1027251-S), and (3) the *Institut de la statistique du Québec*. All COPE Cohort participants provided free and informed consent (studies conducted using self-reported questionnaire data only and a portion for the linkage with provincial health administrative databases).

### Study variables

#### Frequent medical care

Using health administrative databases, we calculated the number of all-cause medical visits in the year following the date of the questionnaire completion (index date). Quebec health administrative databases allow for the tracking of all interactions with physicians in a fee-for-service billing context. For this study, we focused on all medical visits recorded in the fee-for-service medical services database, i.e., outpatient visits, including those in primary care clinics and specialized outpatient clinics, in addition to emergency department visits. Studies on frequent healthcare utilization often focus on emergency department visits only [22, 48]. However, we chose to include all medical visits because it provided a more comprehensive understanding of healthcare utilization patterns. Hospitalizations were not considered, as they are not included in the fee-for-service medical services database and were, moreover, rare in our study population.

The literature provides various threshold-based definitions for frequent healthcare users [17], ranging from 3 [49] to 25 visits per year [50]. Other definitions employ number of visits percentiles [20] or cost-based measure [24] to categorize frequent users. In our study, we defined frequent users as those in the top 10% (90th percentile) of our sample, based on the number of outpatient medical visits during the year following the index date. This approach, used in several studies [20, 48, 51], captures the distinct characteristics of our sample, acknowledging that the significance of 3 visits per year may differ across different study populations. This definition is consistent with the one employed by Canadian agencies like the Canadian Institute for Health Information (CIHI) [52], facilitating comparisons.

#### Sex at birth

Sex assigned at birth was obtained from the health administrative databases, where the available categories were male and female. This variable is considered robust, as it was only in 2022 that Quebec residents became eligible to request a change of sex designation on their birth certificates, including the non-binary option “X.” As of 2024, the provincial government also authorized the use of “X” on health insurance cards and driver’s licenses [53, 54]. In other words, this change does not affect our project, which is based on data collected up to 2020.

#### Gender identity

Gender identity refers to how individuals perceive themselves and identify as men, women, non-binary, gender fluid, two-spirit, and other identities [27]. Distinct from the sex assigned at birth, it provides valuable insights into the psychosocial factors influencing treatment use [55]. Surveys from Canada, the United States, and Europe indicate that 97% of people report a gender identity that aligns with their sex assigned at birth [56, 57]. In Canada, approximately 100,800 persons (0.24%) identify as transgender and non-binary [58]. In the COPE questionnaire, participants were asked to specify their gender as woman, man, indeterminate, or unknown. This question was adapted from the National Institutes of Health (NIH) Task Force on Research Standards for Chronic Low Back Pain’s minimal data set published in 2014 [42] and translated into Canadian French in 2017 [59]. For analysis, the categories “indeterminate” and “unknown” were combined under the term “non-binary,” reflecting a more inclusive term (an umbrella term covering all gender identities that do not fit within the men/women binary) [60]. Given our access to both sex at birth (from administrative data) and current gender identity (from the questionnaire), we were able to identify transgender participants, i.e., those whose gender identity did not align with the sex they were assigned at birth. Non-binary and transgender individuals were grouped under the category “gender-diverse” in our analysis.

#### Gender-stereotyped personality traits

In the COPE Cohort, gender-stereotyped personality traits were assessed using a validated French adaptation of the Bem Sex-Role Inventory (BSRI) consisting of 18 items [61, 62]. The BSRI is a validated tool originally comprising 60 items [63], with several shorter versions available, including the one employed in this study [62]. This French adaptation was chosen because it is brief and considered appropriate for individuals with different literacy levels (items are understood by adolescents) [62]. Each item was scored on a 7-point Likert scale (1 = never true; 7 = always true) [61]. Items (10 items for the feminine subscale and 8 for the masculine subscale) were averaged to obtain a feminine subscale score (0 to 70) and a masculine subscale score (0 to 56) [62]. These 2 scores can be used as continuous variables, or combined for the creation of four subgroups using the split median approach [64, 65]: (1) stereotypically feminine traits, (2) stereotypically masculine traits, (3) androgynous traits, and (4) undifferentiated traits. Specifically, those categorized as “feminine” tend to describe themselves as tender and sensitive to others; those categorized as “masculine” describe themselves as athletic, with leadership and self-confidence; those categorized as “androgynous” score high on all these traits, and “undifferentiated” individuals score low on all traits. Thus, classification should be interpreted accordingly. In our COPE Cohort sample, the internal consistency and factor structure of Fontayne et al.’s short version of the BSRI were found to be adequate [66], i.e., Cronbach’s alphas of 0.90 [95% confidence interval (95% CI) = 0.89–0.91] and 0.82 (95% CI = 0.81–0.84) were obtained for the feminine and masculine scales respectively; confirmatory factor analysis reproduced the five first-order factors (tenderness, sensitivity to others, athletic, leadership, self-confidence) and two second-order factors (feminine, masculine) of the theoretical model published by Fontayne et al. (2000) with acceptable goodness of fit indices; χ^2^ [67] = 1202.62, *p* < 0.0001, GFI = 0.9008, CFI = 0.9147, RMSEA = 0.0823) [66]. Although criticized by some authors (e.g., femininity and masculinity vary across cultures and eras [63]), the BSRI remains the most widely used validated instrument for assessing traits beyond gender identity and has been validated in various contexts [68].

#### Covariables

In addition to sex and gender-related variables, other sociodemographic characteristics measured in the questionnaire were: age (continuous), country of birth (Canada vs. outside of Canada), indigenous and race self-identification (Statistics Canada’s Canadian Community Health Survey questions [69]), employment status (full-time, part- time, unemployed), highest level of education completed, and region of residence (six of the seventeen regions of the province are qualified as remote resource regions by the Quebec government [69] which allowed such a classification). Self-reported pain characteristics included: pain duration (years), pain intensity (0–10 numerical rating scale measuring the average pain intensity in the last 7 days, classified as mild, moderate and severe [70]), various pain locations as dichotomous variables (yes/no), multisite pain (defined as ≥ 2 sites), presence of generalized pain, and pain frequency (continuous or occasional). Catastrophic thinking was assessed by agreeing/disagreeing with the following statement “*I feel that my pain is terrible and it’s never going to get any better*”. This single item from the Pain Catastrophizing Scale [71] is referred to as “catastrophizing” in the National Institute of Health minimal dataset for chronic low back pain [42] and in the STarT Back Screening Tool [72]. Neuropathic pain was evaluated using the validated DN4 Interview part (a score of ≥ 3/7 indicates a likely presence of neuropathic pain) [73], and pain interference using the Brief Pain Inventory (BPI) Interference Scale [74], which ranges from 0 to 10. Self-reported treatment and health status variables included: pharmacological pain treatment use (over-the-counter medications, prescribed medications), perceived general health (one item from SF-12 [75]), percentages of pain relief (participants reaching substantial pain relief, i.e., 50% [76]), excessive polypharmacy (defined as the concurrent use of ≥ 10 medications for pain or other health problems), side effects associated with medications, use of cannabis for pain management, physical and/or psychological treatments use (combined in one category as CP is a disease of biopsychosocial origin and treatments can have an impact on both physical and psychological components at the same time), and access to a trusted healthcare professional for pain management. The questionnaire also inquired about feeling the need to reduce alcohol or drug consumption (never, rarely, sometimes, often consumed more than you meant to), smoking habits (smokers, not a smoker, smoked in the past), and psychological distress using the validated Patient Health Questionnaire 4-item (PHQ-4) [77]. Variables determined using health administrative data (1-year time window before questionnaire completion) included public/private prescription drug insurance status, and a comorbidity score (combined Charlson [78] & Elixhauser [79] index with the International Classification of Diseases 9th revision (ICD-9). This score evaluates the presence and severity of multiple health conditions, with higher scores indicating greater risk of complications and mortality [80, 81].

### Statistical analysis

Descriptive statistics, i.e., means, standard deviations, medians, interquartile ranges (IQR), counts, and percentages, were used to summarize the study population characteristics. A graph was also used to display the distribution of gender personality traits across gender identities. Descriptive statistics were used to describe medical care utilization in terms of annual number of outpatient medical visits and proportion of frequent medical care users (our main dependant variable). To better capture the clinical profile of frequent vs. non-frequent medical care users, descriptive statistics accompanied by bivariable tests were conducted to compare pain intensity, pain interference, perceived general health, percentages of pain relief, excessive polypharmacy, and comorbidity scores between groups (Chi-square for proportions and Wilcoxon rank-sum test for continuous variables). Annual number of outpatient medical visits and proportion of frequent medical care users were then compared across subgroups defined by our three main independent variables, i.e., sex at birth, gender identity, and gender-stereotyped personality traits (Chi-square tests with Tukey-style multiple comparisons of proportions for post-hoc pairwise analyses and Kruskal-Wallis test with Dwass-Steel-Critchlow-Fligner test for post-hoc pairwise analyses).

#### Sex- and gender-based analysis plus

Sex- and gender-based analysis (SGBA) is an approach that systematically examines sex-based (biological) and gender-based (socio-cultural) differences between men, women, boys, girls, and gender-diverse people [27, 82]. Sex and gender are not independent of other characteristics such as age, race, education, as they can interact with each other and with other characteristics to influence health outcomes, as indicated by the “plus” factor of SGBA+ [83]. These other sociodemographic characteristics are important to understand the impact of sex and gender on medical visits, and consider individuals holistically [26, 84]. In contexts where multiple social determinants of health intersect to create inequities [85], and where testing a large number of interactions in a multivariable model becomes challenging, it is valuable to explore alternative statistical approaches that account for intersection factors [25, 86, 87].

In our analysis, we have chosen to form intersecting sociodemographic subgroups based on a cluster analysis and then use this classification as the independent variable in a multivariable logistic regression model built to understand the associations between sex, gender, and frequent medical care utilization. Such an approach is recommended among strategies to incorporate intersecting variables into statistical analyses [86]. Cluster analysis was chosen over other cross-sectional group-based methods (e.g., latent class analysis) as it does not assume independence of observed variables, and is simple to implement (e.g., SPSS^®^ interface that allows users to perform complex statistical analyses without extensive programming) [88]. This approach is an innovative person-centered analysis that identifies groups based on common traits and interrelated theoretical dimensions [89] and was used in previous SGBA + studies [88, 90].

A two-step cluster analysis was used, which allows integration of categorical and continuous variables [91, 92], and incorporated the following intersecting factors: categorical variables included sex at birth, gender identity, residence in a remote region, country of birth, education level, and employment (as socioeconomic status proxy); continuous variables included age and gender-stereotyped personality traits (feminine and masculine scores). The sociodemographic variables to be included in the cluster analysis were selected a priori, following recommendations [5]. The optimal number of intersecting sociodemographic subgroups was determined based on theory (important variables associated with sex and gender [5, 93]) and validity, that is: (1) the Bayesian Information Criterion (BIC) [94], confirmed via graphical assessment and the elbow method (Supplementary content 2) [2, 95, 96], (2) the average silhouette coefficient [97], a measure of how similar individuals are to their cluster compared to other clusters (ranges from − 1 to 1, where values > 0.50 are interpreted as good fitting, values between 0.30 and 0.50 as fair fitting and values < 0.30 as poor fitting [98]), (3) the cluster size ratio (largest to smallest cluster), to verify we have an appropriate number of people in each cluster [99], and (4) adequate interpretability of intersecting sociodemographic subgroups (e.g., to be able to assign meaningful labels to each group by observing the variables for which the groups differed the most). The intersecting sociodemographic subgroups resulting from the cluster analysis then served as independent variables for subsequent analyses.

#### Multivariable analysis

A multivariable logistic regression model was employed to examine the association between intersecting sociodemographic subgroups (independent variable) and frequent medical care utilization (dichotomous dependent variable), adjusting for potential confounders (all the covariables mentioned above). Adjusted odds ratios (ORs) and 95% confidence intervals (CIs) were calculated. The reference category for the creation of intersecting sociodemographic subgroups dummy variables was one of our standout groups, composed almost exclusively of men (97%). All covariables were preselected a priori according to the latest recommendations for multivariable model construction [100]. Selection was based on existing literature, clinical relevance, and the Andersen model [101], which identifies predisposing, enabling, and need factors in healthcare utilization. This method was favoured over criticized selection techniques such as relying on bivariable regression analysis p-values [100] or stepwise selection [102]. Multicollinearity problems were screened (variance inflation factors below 5) [103], and Hosmer-Lemeshow test (*p* > 0.05) supported the goodness of fit of the model. Variables included in the final model were missing completely at random (MCAR), as indicated by the results of Little’s MCAR test (Chi-square = 1.782, DF = 2, *p* = 0.410). Listwise deletion was thus an acceptable approach for our multivariable model [102, 104]. Sensitivity analyses were conducted, including: [1] using unemployed more educated women as the reference subgroup [2], and considering only outpatients medical visits within a 6-month time window following questionnaire completion (to mitigate COVID-19 impact on our results, as a portion of the 12-month time window used to define medical care utilization overlapped the beginning of the first wave of the pandemic in Canada, i.e., march 2020 [105]). All analyses were performed using SPSS Statistics version 29^®^ (IBM Corp, Armonk, NY) and SAS version 9.4^®^ (SAS Institute, Cary, NC).

## Results

### Characteristics of our study population

Among the 1935 COPE Cohort participants, 921 (47.6%) provided consent for data linkage. A total of 895 participants were included in the analysis (those for which the linkage was successful). The study population characteristics are presented in Table 1. The mean age was 50.3 ± 13.2 years, with 82.9% female and 17.1% male. In terms of gender identity, 82.3% identified as women, 16.8% as men, and 0.9% as gender-diverse. Regarding gender-stereotyped personality traits, 23.3% exhibited feminine traits (e.g., tenderness, sensitivity), 18.4% masculine traits (e.g., leadership, athleticism, self-confidence), 32.9% androgynous traits (high scores on both masculine and feminine traits), and 25.4% undifferentiated traits (low scores for both masculine and feminine traits).

**Table 1:** Sample characteristics

Characteristics (*n* = 895)	No. (%) of participants^a^
Sex at birth	
Male	153 (17.1)
Female	742 (82.9)
Gender identity	
Men	150 (16.8)
Women	737 (82.3)
Gender-diverse	8 (0.9)
Gender-stereotyped personality traits (categorized BSRI scores)	
Feminine	183 (23.3)
Masculine	145 (18.4)
Androgynous	259 (32.9)
Undifferentiated	200 (25.4)
Age (years) – mean ± SD	50.3 ± 13.2
Country of birth	
Canada	837 (93.5)
Outside Canada^b^	33 (3.7)
Indigenous self-identification	
Non-Indigenous	842 (94.1)
Indigenous peoples	14 (1.6)
Race self-identification	
White	849 (94.9)
Racialized	16 (1.8)
Employment	
Worker full time	207 (23.8)
Worker part-time	94 (10.8)
Unemployed	568 (65.4)
Education level	
Secondary diploma or below	182 (21.0)
Post-secondary diploma (below university level)	324 (37.5)
University diploma	359 (41.5)
Region of residence	
Remote^c^	203 (23.2)
Non-remote	672 (76.8)
Pain duration (years)	
Over 3 months but less than a year	27 (3.0)
1–4	207 (23.2)
5–9	205 (22.9)
≥ 10	455 (50.9)
Pain intensity in the past 7 days (categorized 0–10 score)	
Mild (1–4)	265 (30.0)
Moderate (5–7)	483 (54.8)
Severe (8–10)	134 (15.2)
Four most common pain locations^d^	
Back	551 (61.6)
Neck	392 (43.8)
Shoulder(s)	391 (43.7)
Leg(s)	356 (39.8)
Pain frequency	
Continuously	793 (88.9)
Occasionally	99 (11.1)
Agreeing with the statement ‘’ I feel that my pain is terrible and it’s never going to get any better’’	
Yes	540 (60.5)
No	353 (39.4)
Pharmacological pain treatments use	
Yes	839 (94.7)
No	47 (5.3)
Physical/psychological pain treatments use	
Yes	742 (82.9)
No	146 (16.3)
Access to a trusted healthcare professional for pain management	
Yes	714 (80.5)
No	173 (19.5)

*SD* Standard deviation; *BSRI* Bem Sex-Role Inventory

^a^Unless stated otherwise

^b^Outside Canada: Algeria, Germany, Belgium, New England, Colombia, Cuba, United States, France, Haiti, Italy, Laos, Lebanon, Morocco, Poland, Democratic Republic of the Congo, Senegal, Switzerland, Venezuela

^c^Remote resource regions as defined by Revenu Quebec (i.e., the provincial revenue agency): Bas-Saint-Laurent, Saguenay—Lac-Saint-Jean, Abitibi-Témiscamingue, Côte-Nord, Nord-du-Québec, Gaspésie—Îles-de-la-Madeleine. Non-remote regions are near a major urban center

^d^Non-mutually exclusive categories. The proportion of missing data across the presented variable ranges between 0 and 12.07%

**Fig. 2:**
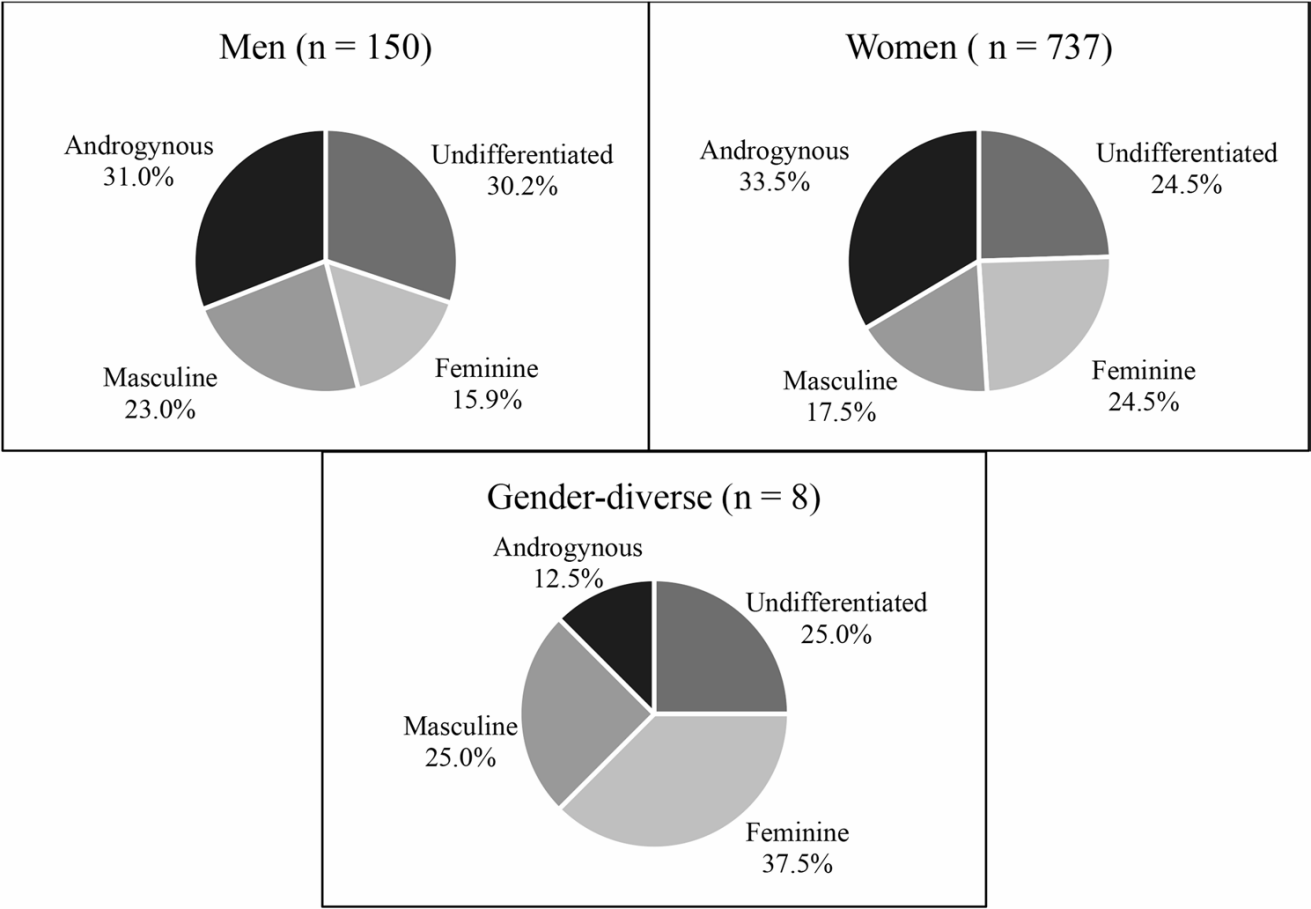
Distribution (%) of gender-stereotypes personality traits subgroups in men (left), women (right) and gender-diverse persons (middle down)

Figure 2 displays the distribution of gender-stereotyped personality traits across men, women, and gender-diverse persons and shows that these two variables do not completely overlap. Among men, 15.9% exhibited feminine traits, 23.0% masculine traits, 31.0% androgynous traits, and 30.2% undifferentiated traits. Among women, the proportions were 24.5%, 17.5%, 33.5%, and 24.5%, respectively, while among gender-diverse persons, the proportions were 37.5%, 25.0%, 12.5%, and 25.0%. Across all gender identities, personality traits varied, with most men and women displaying androgynous traits, while gender-diverse persons display more feminine traits.

Feminine: described themselves as tender and sensitive to others. Masculine: described themselves as athletic, having leadership, and being self-confident. Androgynous: scored high on all these traits. Undifferentiated: scored low on all these traits.

### Medical care utilization

Table 2 presents the distribution of all-cause medical visits (primary care clinics and specialized outpatient clinics, in addition to emergency department visits) over the year following questionnaire completion. On average, participants had 5.7 ± 5.1 medical visits (median = 4.0, IQR: 2.0–8.0), with most visits made to a family physician. For frequent medical care users, defined as the top 10% of the sample, 95 participants had 13 or more medical visits within the year. Among this group, the mean number of medical visits was 16.6 ± 4.7 medical visits (median = 15.0, IQR: 15.0–18.0).

**Table 2:** Descriptive statistics of 1-Year medical visits

1-year medical visits (*n* = 895)	Descriptive statistics
Visits to a family physician - mean ± SD Median (IQR)	4.3 ± 4.1 3.0 (1.0-6.0)
Visit to other medical specialists - mean ± SD Median (IQR)	1.3 ± 2.7 0.0 (0.0-2.0)
Emergency visit - mean ± SD Median (IQR)	0.65 ± 1.29 0.0 (0.0-1.0)
Total medical visits - mean ± SD Median (IQR)	5.7 ± 5.1 4.0 (2.0-8.0)
Minimum	0
Maximum	37
90^th^ Percentile	13
Frequent medical care utilization - *n* (%)	95 (10.6)

Frequent medical care users are defined as participants with ≥ 13 visits per year (90th percentile)

*SD* Standard deviation, *IQR* Interquartile range

Frequent medical care users exhibited a different clinical profile compared to non-frequent users (*p* < 0.05). As shown in Table 3, frequent users reported a greater pain interference, a poorer perceived general health, were less likely to ≥ 50% pain relief, and were more likely to report excessive polypharmacy (≥ 10 medications).

**Table 3:** Clinical profile of frequent vs. non-frequent medical care users

	Frequent medical care users (*n* = 95)	Non-frequent medical care users (*n* = 800)	*p* – value
Pain intensity in the last 7 days (score 0–10) – n (%)			
Mild (1–4)	22 (23.2)	243 (30.9)	0.184
Moderate (5–7)	54 (56.8)	429 (54.5)	
Severe (8–10)	19 (20.0)	115 (14.6)	
Pain interference (BPI score) – mean ± SD	6.53 ± 1.87	5.61 ± 2.22	**< 0.001**
Perceived general health (item from SF-12) – n (%)			
Excellent	2 (2.2)	21 (2.8)	**0.030**
Very good	7 (7.7)	95 (12.6)	
Good	23 (25.3)	247 (32.7)	
Fair	27 (29.7)	236 (31.2)	
Poor	32 (35.2)	157 (20.8)	
Percentage of pain relief ≥ 50% – n (%)			
No	56 (59.6)	360 (45.9)	**0.012**
Yes	38 (40.4)	424 (54.1)	
Excessive polypharmacy (≥ 10 medications) – n (%)			
No	55 (60.4)	545 (72.4)	**0.018**
Yes	36 (39.6)	208 (27.6)	
Comorbidity score (Charlson & Elixhauser index) – mean ± SD	0.09 ± 0.49	0.13 ± 0.69	0.592

Frequent medical care users are defined as participants with ≥ 13 visits per year (90th percentile). The proportion of missing data across the presented variable ranges between 0 and 29.1%. p-values < 0.05 are reported in **bold**

*SD* Standard deviation, *BPI* Brief pain inventory, *SF *12:12-item Short Form Survey

## Medical care utilization across sex and gender subgroups

Table 4 presents the mean number of medical visits and the proportion of frequent medical care users across sex, gender identity, and gender-stereotyped personality traits. Compared to males, females were more likely to be frequent medical care users (12.0% vs. 3.9%; *p* = 0.003). When looking at gender identity, a higher proportion of individuals identifying as women were frequent medical care users compared to men and gender-diverse persons (12.1% vs. 4.0% vs. 0.0%; *p* = 0.009). No differences (*p* > 0.05) were observed between subgroups defined by gender-stereotyped personality traits.

**Table 4:** Medical visits across sex at birth, gender identity and gender-personality traits

	Annual number of medical visits mean ± SD (median; IQR)	*p*-value	Frequent medical care users *n* (%)	*p*-value
Sex at birth				
Male (*n* = 153)	4.7 ± 4.2 (4.0; 2.0–7.0)	0.141	6 (3.9)	** 0.003**
Female (*n* = 742)	5.9 ± 5.3 (4.0; 2.0–8.0)		89 (12.0)	
Gender identity				
Men (*n* = 150)	4.7 ± 4.2 (4.0; 2.0–7.0)	0.225	6 (4.0)	**0.009**
Women (*n* = 737)	5.9 ± 5.3 (4.0; 2.0–8.0)		89 (12.1)	
Gender-diverse (*n* = 8)	5.8 ± 2.7 (6.0; 3.5–7.5)		0 (0.0)	
Gender-stereotyped personality traits				
Feminine (*n* = 183)	6.2 ± 5.1 (5.0; 2.0-8.5)	0.282	25 (13.7)	0.429
Masculine (*n* = 145)	5.5 ± 5.0 (4.0; 2.0–9.0)		14 (9.7)	
Androgynous (*n* = 259)	5.2 ± 4.5 (4.0; 2.0–7.0)		23 (8.9)	
Undifferentiated (*n* = 200)	5.9 ± 6.0 (4.0; 2.0–8.0)		22 (11.0)	

*p*-values < 0.05 are reported in bold**.** The proportion of missing data across the presented variable ranges between 0 and 16.0%.

*SD* Standard deviation; *BSRI* Bem sex-role inventory, *IQR* Interquartile range

### Intersecting sociodemographic subgroups

**Fig. 3:**
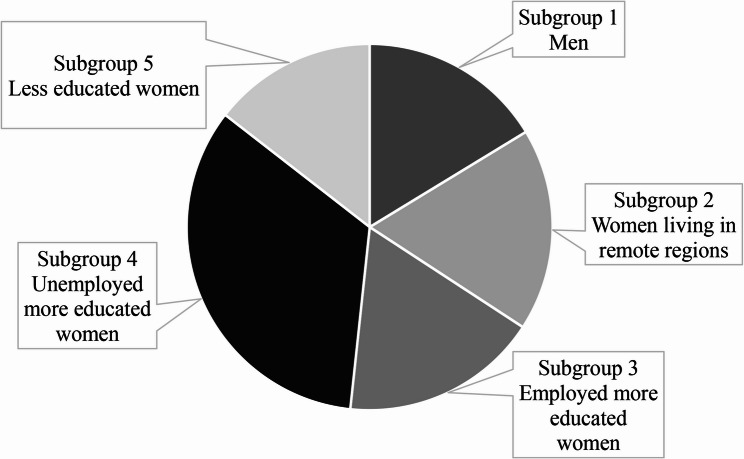
Intersecting sociodemographic subgroups with specific labels

The elbow method indicated an optimal fit for a number of clusters between 2 and 7, with the best fit observed for 6 to 7 clusters (lower BIC values) (Supplementary content 2). As suggested by some authors [88, 106], we opted for a clustering solution that offers both adequate statistical fit and yields interpretable clusters with clear qualitative distinctions. Based on these criteria of rigor and interpretability, a five-cluster solution was deemed ideal. The silhouette coefficient (SC = 0.4) and the cluster size ratio (2.33) were fair. Each intersecting sociodemographic subgroup represented more than 5% of the sample (minimum 30 observations per group) [107], and the subgroups exhibited good interpretability. According to the cluster analysis, 5 variables appeared to have more influence on the group’s classification (sex at birth, gender identity, living in a remote region, education, and employment). The final five intersecting sociodemographic subgroups were labelled as follows: subgroup 1 ‘*Me*n’, subgroup 2 ‘*Women living in remote regions*’, subgroup 3 ‘*Employed more educated women*’, subgroup 4 ‘*Unemployed more educated women’*, and subgroup 5 ‘*Less educated women’* (Fig. 3). Discriminant short labels were given to each subgroup, but the complete characteristics of each subgroup can be found in Table 5. For example, a men-only label was created, but it was noted that they were mostly unemployed, educated, and living in urban setting.

**Table 5:** Descriptives results of cluster analysis

*n* = 772	Subgroup 1 Men *n* (%)	Subgroup 2 Women living in remote regions *n* (%)	Subgroup 3 Employed more educated women *n* (%)	Subgroup 4 Unemployed more educated women *n* (%)	Subgroup 5 Less educated women *n* (%)
Number and proportion of the cohort in each subgroup	126 (16.3)	138 (17.9)	135 (17.5)	261 (33.8)	112 (14.5)
Sex at birth					
Female	1 (0.8)	138 (100.0)	135 (100.0)	261 (100.0)	112 (100.0)
Male	125 (99.2)	0 (0.0)	0 (0.0)	0 (0.0)	0 (0.0)
Gender identity					
Women	0 (0.0)	137 (99.3)	134 (99.3)	260 (99.6)	111 (99.1)
Men	122 (96.8)	(0.0)	0 (0.0)	0 (0.0)	0 (0.0)
Gender-diverse	4 (3.2)	1 (0.7)	1 (0.7)	1 (0.4)	1 (0.9)
Gender-stereotyped personality traits (BSRI continuous scores)** –** mean ± SD					
Feminine	5.4 ± 1.1	5.7 ± 0.8	5.8 ± 1.0	5.7 ± 0.9	5.7 ± 1.0
Masculine	3.9 ± 1.2	4.0 ± 1.1	3.9 ± 1.1	3.8 ± 1.1	3.4 ± 1.2
Age (years)** –** mean ± SD	55.6 ± 13.2	45.9 ± 12.7	42.6 ± 10.9	52.0 ± 13.0	51.7 ± 11.8
Country of birth					
Canada	121 (96.0)	137 (99.3)	129 (95.6)	245 (94.2)	109 (98.2)
Outside Canada^a^	5 (4.0)	1 (0.7)	6 (4.4)	15 (5.8)	2 (1.8)
Employment					
Worker full time	35 (27.8)	84 (60.9)	135 (100.0)	2 (0.8)	19 (17.0)
Unemployed	91 (72.2)	54 (39.1)	0 (0.0)	259 (99.2)	93 (83.0)
Education level					
Post-secondary education	99 (78.6)	123 (89.1)	135 (100.0)	261 (100.0)	0 (0.0)
No post-secondary education	27 (21.4)	15 (10.9)	0 (0.0)	0 (0.0)	112 (100.0)
Region of residence					
Remote^b^	25 (19.8)	138 (100.0)	0 (0.0)	261 (100.0)	16 (14.3)
Urban	101 (80.2	0 (0.0)	135 (100.0)	261 (100.0)	96 (85.7)

The following variables were used to realize the cluster analysis: age, sex at birth, gender identity, gender personality traits according to the BSRI, country of birth, employment, education level, region of residence

*BSRI* Bem Sex Role Inventory

^a^Outside Canada: Algeria, Germany, Belgium, New England, Colombia, Cuba, United States, France, Haiti, Italy, Laos, Lebanon, Morocco, Poland, Democratic Republic of Congo, Senegal, Switzerland, Venezuela

^b^Remote resource regions as defined by Revenu Quebec (i.e., the provincial revenue agency): Bas-Saint-Laurent, Saguenay—Lac-Saint-Jean, Abitibi-Témiscamingue, Côte-Nord, Nord-du-Québec, Gaspésie—Îles-de-la-Madeleine. Non-remote regions are near a major urban center

### Intersecting sociodemographic subgroups as a determinant of frequent medical care utilization

**Fig. 4:**
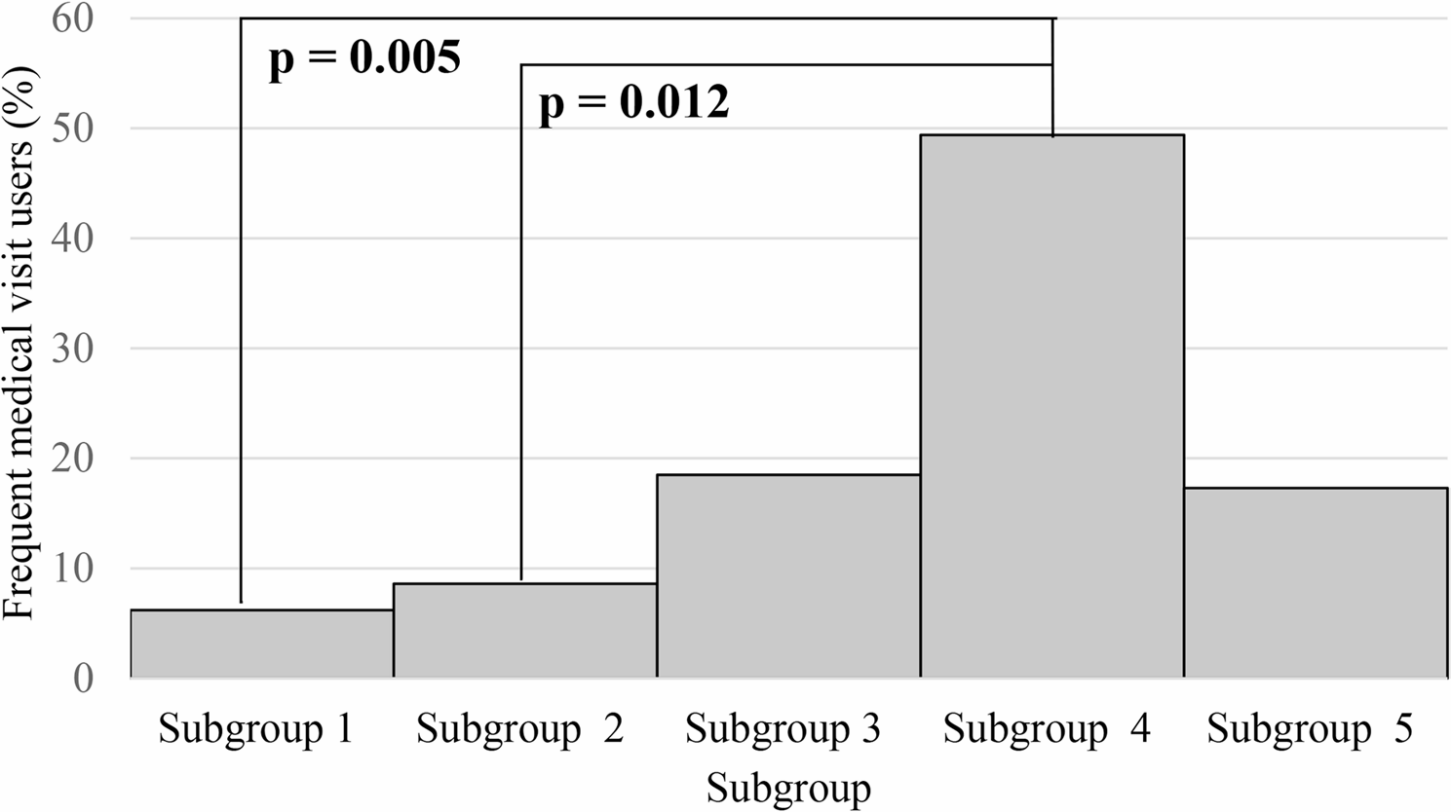
Proportion of frequent medical care users across intersecting sociodemographic subgroups

Proportion of frequent medical care users across intersecting sociodemographic subgroups is presented in Fig. 4. The bivariable analysis revealed statistically significant differences (*p* < 0.05) between ‘*Unemployed more educated women*’ and ‘*Women living in remote regions*’, as well as between ‘*Unemployed more educated women’* and ‘*Men*’ subgroups.

The line indicates a statistically significant difference (*p* < 0.05) between the two corresponding groups (post hoc Tukey-style multiple comparisons of proportions test).

Table 6 presents the key findings from the multivariable logistic regression model assessing the association between intersecting sociodemographic subgroups and frequent medical care utilization. After adjusting for potential confounders, the model indicated that membership to the ‘*Unemployed more educated women*’ subgroup, as compared to the ‘*Men*’ subgroup, significantly increases the odds of being a frequent medical care user (OR: 3.93; 95% CI: 1.44–10.80, *p* = 0.01). Sensitivity analyses confirmed the stability of this conclusion when using the ‘*Unemployed more educated women*’ subgroup as the reference category or when using a 6-month time window for the assessment of frequent medical care utilization (meaning no COVID-19 impact on our results). In the multivariable model, covariables associated with frequent medical visits utilization were greater pain interference (OR: 1.38; 95% CI: 1.15–1.67, *p* < 0.001) and access to a trusted healthcare professional for pain management (OR: 2.94; 95% CI: 1.18–7.30, *p* = 0.02). Complete multivariable results are provided in Supplementary Content 3.

**Table 6:** Multivariable model exploring associations between intersecting sociodemographic subgroups and frequent medical care utilization

Variable	Odds ratio (Adjusted OR)	*p*-value	95% CI
Intersecting sociodemographic subgroups (reference: ‘Men’)			
‘Women living in remote regions’	1.25	0.72	0.37–4.30
‘Employed more educated women’	2.50	0.11	0.80–7.75
‘Unemployed more educated women’	** 3.93**	**0.01**	**1.44–10.80**
‘Less educated women’	2.98	0.06	0.94–9.47

Complete multivariable results are provided in Supplementary Content 3

*p*-values < 0.05 are reported in bold, 95% CI: 95% confidence interval and OR: odds ratio.The multivariable analysis was adjusted for the following covariables: pain duration, pain intensity in the last 7 days, multisite pain, generalized pain, pain frequency, agreeing with the statement ‘’I feel that my pain is terrible and it’s never going the get any better’’, evidence of neuropathic pain according to the DN4 scale, pain interference according to the BPI score, pharmacological pain treatments use, excessive polypharmacy (≥10 medications), side effects associated with medication, use of cannabis for pain management, physical/psychological pain treatments use, access to a trusted health care professional for pain management, feeling the need to reduce alcohol or drug consumption, smoking habits, psychological distress according to the PHQ scale, having private prescription drug insurance, and comorbidity score (Charlson & Elixhauser index). In total, 717 participants (80.1%) were included in the final model (178 missing data; 19.9% were excluded)

## Discussion

We conducted an innovative analysis incorporating sex, gender, and their intersection with other social determinants of health in a diverse cohort of persons living with CP. Our study revealed that frequent medical care users exhibit a more severe clinical profile, characterized by a poorer perceived general health, fewer chances of achieving substantial pain relief, greater pain interference, and greater percentage of excessive polypharmacy. Women, particularly those who are unemployed and more educated, were more likely to be frequent medical care users.

### Frequent medical care utilization

CP, often characterized by a complex clinical profile, is one of the most common reasons for seeking healthcare [15]. Persons living with CP are more likely to be frequent users of healthcare services [20, 21]. When looking at the average of 13 medical visits per year, it raises the question of whether this number of consultations is excessive or appropriate. In comparison with the general Canadian population, the average number of physician visits per person per year ranges from 4.8 to 6.1 visits [108, 109]. Based on our study, frequent medical care visits are mostly associated with a worse health profile (in terms of general health, pain interference and relief, and polypharmacy). Other studies among the general population reported that frequent healthcare users are more at risk for incapacity, poorer quality of life, mortality [110–113], and poorer mental health [17]. In CP studies, frequent healthcare utilization was also associated with worse health profiles such as higher depressive symptoms [114], greater pain interference or pain-related disability [21, 24], low pain self-efficacy, and more comorbidities [115]. As unfavourable health profiles seem to be associated with frequent healthcare utilization, it is important to better support frequent users. Aiming at more efficient healthcare system navigation, interventions such as the integration of case management, i.e., a collaborative process involving assessment, planning, facilitation, care coordination, evaluation, and advocacy to address an individual’s and family’s comprehensive health needs [116], have proven effective for frequent healthcare users [117]. Individualized care plans and information sharing also seem to help reduce medical use [117].

### Sex, gender and frequent medical care use in chronic pain populations

Understanding the sociodemographic characteristics of frequent users is important for identifying those most at risk of unfavourable health profiles. Our bivariable analysis revealed that women were more likely to be frequent medical care users compared to men and gender-diverse persons. Generally, it is acknowledged that women are more likely to seek treatment [19, 118]. Also, gender roles can influence healthcare utilization (e.g., caregiving roles, time for appointments) [119, 120]. Currently, only a few studies have examined the relationship between sex and the frequent healthcare utilization [21, 24]. Gender has never been studied in this context. However, we can draw parallels between our findings and those of studies that have explored the relationship between sex or gender, and the number of healthcare visits [20, 114, 121–125]. Some studies, focusing solely on biological sex, found no significant sex differences in healthcare utilization [67, 114], while others did [20, 122, 124, 125]. A study exploring both sex and gender differences found that sex, but not gender, influenced healthcare utilization patterns [121].

Using an SGBA + analysis, we gained a more nuanced understanding of which women are more likely to be frequent medical care users. Specifically, ‘*unemployed more educated women’* (compared to men) appear to be more likely. It is established that unemployment (regardless of the level of education) can have an impact on persons living with CP [126], and conversely, CP can also impact one’s ability to maintain employment [127]. In a previous study, unemployed individuals (adjusting for age, sex and comorbidities) were more likely to report greater pain interference [128], which may lead to more frequent medical visits [125]. It was also reported that persons experiencing both CP and unemployment report severe physical and emotional impairment, as well as a greater number of healthcare visits [126]. Some hypotheses can be put forward regarding unemployed women being more likely to be frequent medical care users. Socioeconomic factors, e.g., unemployment, are often linked to financial instability, leading individuals to rely more heavily on public healthcare services, which may result in more frequent healthcare consultations [127]. In fact, they often have more limited access to preventive services and alternative therapies offered in the private sector covered by private insurance through employers (e.g., physiotherapy), potentially increasing their utilization of public medical services [127]. Although women rely more often on non-pharmacological treatments to manage their pain compared to men [129], it can be more challenging to access a multimodal treatment approach (a combination of medication with non-pharmacological treatments such as psychotherapy, physiotherapy, acupuncture, etc.) when unemployed, as those therapies are often expensive and only covered by private insurance through employers [130]. Another hypothesis is that frequent healthcare utilization could be associated with a higher likelihood of job loss, a temporality of events that should be explored in future studies. Also, time for medical appointments can possibly be influenced by work status [131]. Regarding the level of education, some studies suggest that higher education is associated with greater healthcare utilization, particularly for specialized treatments [132] and preventive care [133]. Others report the opposite effect, where greater educational attainment is associated with lower use of services, such as fewer emergency visits [134], and greater self-management and self-awareness [133]. It is also reported that education appears to have stronger health effects for women than men [135]. Now from an SGBA + perspective, women who are both unemployed and educated may face unique challenges. While their education equips them with greater health literacy and expectations [136, 137], their unemployment status may create a mismatch between aspiration and lived realities, leading to increased health-seeking behaviors [138]. Overall, in our study, being a woman, more educated and being unemployed was associated with the use of medical services and we put forward that it is not simply an addition of factors, but rather an intersection of multiple potential challenges.

In our study, region of residence did not emerge as a predictor of frequent medical visits, which may be explained by our analytical strategy. Rather than being included as an individual independent variable in the multivariable model, region of residence was incorporated into the creation of intersecting sociodemographic subgroups (cluster analysis). One subgroup, i.e., ‘Women living in remote regions’, initially appeared distinct during the subgrouping process; however, it did not remain statistically different from other groups in the multivariable analyses. These results suggest the need for further studies focusing specifically on the role of geographic region in frequent healthcare utilization among individuals living with CP.

### Recommendations

Based on our results, we can underline the need for clinicians and decision-makers to adopt an integrated approach considering both clinical and socioeconomic profiles for effective healthcare. Understanding a patient’s socioeconomic situation enables better guidance toward appropriate care, aligned with their resources [139]. For instance, unemployed women without private insurance should be directed to accessible services, such as community-based programs or low-cost support options like public rehabilitation or affordable psychological services or support groups. Emphasizing self-management through free programs could also be beneficial [140]. Many free self-management programs are available [141, 142]. A more tailored approach, considering patients’ sociocultural context is warranted [117], along with addressing gender inequalities in CP, and advocating for inclusive and diverse care [7, 143]. Recommendations such as providing safe spaces for patients and educating employers about health inequalities are interesting options [144].

### Methodological recommendations

A comprehensive understanding of health disparities related to sex and gender requires recognizing the complex interaction between biological sex, gender, and broader societal, cultural, and economic factors [26]. Although sex and gender are distinct, their close interconnection often complicates quantitative analyses [93]. Statistical strategies now enable the integration of intersecting factors into quantitative analyses [5, 25, 86, 87], providing a framework to examine how overlapping identities—such as gender, race, ethnicity, socioeconomic status, age, religion, and disability—shape individual experiences and societal participation [145]. Once significant differences between sex or gender are identified in bivariable analysis, further investigation into the underlying contributing factors is essential. It is important to integrate SGBA+ and adopt a intersectional lens [27, 83, 145] in studies focused on sex and gender. In this study, cluster analysis effectively explored the intersection of sex and gender as predictors of medical care utilization. Interdisciplinary approaches examining the non-binary dimensions of sex and gender, alongside their intersections with social, economic, and cultural factors, will improve our understanding of health disparities, inform targeted interventions, and contribute to healthcare equity [26, 146].

### Strengths and limitations

Our study has several strengths, including a large, province-wide sample of participants from Quebec, Canada. Our sample is representative of probabilistic samples of Canadians living with CP in terms of age, employment status, education level, and pain characteristics [31]. Self-reported data, the gold standard in pain research [147], was collected according to best practices and established recommendations and validated measurement tools were used [41, 45, 59, 148, 149].

Our sample allowed us to study individuals across a broad spectrum of gender-stereotyped personality traits (Fig. 2). Even though the BSRI is a widely validated and used tool [68], it has notable limitations. Since societal attitudes, roles and expectations related to gender have evolved significantly in recent years, it may not fully capture current gender constructs [63]. Other gender-related concepts could be included in future studies to further explore the gender differences, such as gender relations (e.g., marital status) or gender roles (e.g., household responsibilities, caregiving or parental responsibilities, etc.) [150]. The small number of gender-diverse participants (*n* = 8) is another limitation of our study. Also, the intersecting profile did not include Indigenous or racial self-identification due to the small number of such participants in our sample. Further research could benefit from oversampling individuals from these underrepresented groups to explore their experiences more thoroughly.

It should be noted that our study does not allow us to confirm that each medical visit was specifically for CP. The rationale for this approach is that diagnostic codes (ICD-9 and ICD-10) found in health administrative databases are not always valid for identifying cases of CP [151]. This is why we chose to focus on all-cause medical visits. This perspective is still relevant given that CP is frequently associated with multiple chronic comorbidities. By analyzing all-cause medical visits, we capture the overall healthcare experience. This broader lens is relevant when seeking to better understand and support frequent users of healthcare services. For the creation of the sample, linkage between self-reported and longitudinal health administrative data circumvented the difficulty in identifying CP cases when using administrative data only.

## Conclusion

When examining the associations between sex, gender, and frequent healthcare utilization, the use of other sociodemographic variables is informative. It has allowed us to understand that unemployed more educated women living with CP were more likely to be frequent medical care users as compared to other profiles of women or men. Regardless of the potential bidirectional temporality of unemployment and frequent medical care utilization, our results underline the importance of adopting personalized care approaches for frequent medical visit users. Tailored treatment plans should be offered to these patients, e.g., self-management options and community-based care could be recommended by clinicians to help reduce medical visits for this patient group.

## Supplementary Information

Supplementary Material 1.

## Data Availability

The COPE Cohort self-reported data is not readily available because participants did not initially provide consent to open data. Self-reported data are available from the corresponding author upon reasonable request and conditionally to a proper ethical approval for a secondary data analysis. As for health administrative databases, access must be granted by the Institut de la statistique du Québec (ISQ) (data holder). Programming codes can be obtained directly from the corresponding author.

## Abbreviations

BPI: Brief Pain Inventory
BSRI: Bem Sex-Role Inventory
CIHI: Canadian Institute for Health Information
CI: Confidence intervals
COPE: ChrOnic Pain trEatment cohort
CP: Chronic pain
CPN: Chronic Pain Network
ICD-9: International Classification of Diseases 9th revision
IQR: Interquartile ranges
IMMPACT: Methods, Measurement and Pain Assessment in Clinical Trials
ISQ: Institut de la Statistique du Québec
NHI: National Institutes of Health
OR: Odds ratios
PHQ-4: Patient Health Questionnaire 4-item
RAMQ: Régie de l’assurance maladie du Québec
SD: Standard deviation
SF-12: Short-Form Health Survey
SGBA: Sex-and gender-based analysis
SGBA+: Sex-and gender-based analysis plus
SPOR: Strategy for Patient-Oriented Research
