# Supplementary material for: A sex- and gender-based analysis plus of frequent healthcare utilization among individuals living with chronic pain: a cohort study
Source: BMC Health Serv Res. 2025 Oct 1;25:1261. doi: 10.1186/s12913-025-13374-5 (PMC12487626; doi:10.1186/s12913-025-13374-5)
Supplement: Supplementary file 1 — Supplementary Material 1. [file 12913_2025_13374_MOESM1_ESM.pdf]

Supplementary content 1 – COPE Cohort questionnaire

Lacasse, A., Gagnon, V., Nguena Nguefack, H. L., Gosselin, M., Pagé, M. G., Blais, L., & Guénette, L. (2021). Chronic pain patients' willingness to share personal identifiers on the web for the linkage of medico-administrative claims and patient-reported data: The chronic pain treatment cohort. *Pharmacoepidemiology and drug safety*, 30(8), 1012–1026. https://doi.org/10.1002/pds.5255

Description of variables and validated measurement scales included in the COPE Cohort web-based questionnaire.

**Variables**	**Measurement**	**Sections of the questionnaire**
Chronic pain characteristics and interference †		
Location	- Semi closed-ended question *	1
Circumstances surrounding onset	- Semi closed-ended question *	1
Duration	- Open-ended question about the number of days, months or years since the onset of pain (allowing the assessment of chronic pain presence) *	1
Frequency	- Closed-ended question *	1
Intensity	- 11-point (i.e., 0–10) numerical rating scales ‡ – On average in the past 7 days/At its worst in the past 7 days *	1
Tendency to pain catastrophizing	- Closed-ended question : Agreement with the statement "*I feel that my pain is terrible and it's never going to get any better*" §	1
Neuropathic component	- Neuropathic pain questionnaire (DN4) – Interview part (Bouhassira et al., 2005) (a score >3/7 indicates a likely presence of a neuropathic component to the patient's pain; when some items were filled (yes=1 point), items not filled in were considered as 0 points (Timmerman et al., 2017)) *	2
Interference	- Brief Pain Inventory (BPI) – Interference scale (Cleeland, 2009) *‡	3
Pain treatment and healthcare		
Section of the questionnaire designed to obtain consent for data linkage	- Free and informed consent form specific to data linkage (yes/no, reasons of refusal)<br>- Space to provide personal identifiers if linkage with public medical/drug insurance claims (health insurance number, first name, last name, date of birth)<br>- Private drug insurance information if applicable	4
Pharmacological pain treatments	- Closed-ended questions about current use of prescribed pain medications (yes/no) and over-the-counter pain medications (yes/no).<br>- Will be complemented by prescription claims.	5

Adverse effects of pharmacological pain treatments †	- Standardized checklist of adverse effects related to pain treatment evaluated in terms of presence and intensity (mild, moderate, severe) ‖	5
Non-pharmacological pain treatments	- Closed-ended question about current use of non-pharmacological pain treatments (yes/no)<br>- Semi closed-ended question about the type of treatments that are used. Listed treatments were inspired by the work of the Canadian Agency for Drugs and Technologies in Health (CATH, 2018) and the Quebec Pain Registry (Choiniere et al., 2017)	5
Percentage of relief provided by pain treatments	- Numeric scale ranging from 0% (no relief) to 100% (complete relief) adapted from the Brief Pain Inventory (BPI) (Cleeland, 2009). The BPI version only covered the past 24 hours so it was adapted to cover general relief provided by current use of pain treatments.	5
Most effective treatment	- Open-ended question	5
Access to a trusted healthcare professional for pain management	- Closed-ended question with the following examples: physician, nurse, pharmacist, physiotherapist, psychologist	5
Sociodemographic profile		
Age *, gender identity *, race/ethnicity, country of birth, employment *, involvement in litigation related to a disability benefit claim, education level, region of residence	- Open-ended, closed-ended and semi closed-ended questions	6
Gender (gender-stereotyped personality traits) *	- Gender roles scale: Bem Sex-Role Inventory (BSRI) (Bem, 1974) – 18-item French version (Fontayne et al., 2000)	6
Health profile		
Health-related quality of life †	- 3 items of the SF-12v2 Health Survey (SF-12v2) (Maruish, 2012) allowing the norm-based scoring of 2 of the 8 SF-12v2 subscales, i.e., Physical Functioning (PF) * and General Health (GH)	7
Polypharmacy	- Closed-ended question about the number of medications currently used (including prescribed, over-the-counter, pain-related and other diseases-related medications)	7
Emotional functioning †	- Anxiety and depressive symptoms measured by the Patient Health Questionnaire - 4 items (PHQ-4) (Kroenke et al., 2009) *	7

Smoking, alcohol and drugs	- Closed-ended questions §	7
Cannabis use	- Closed-ended questions about past year use of cannabis for pain management (yes/no), management of other health problems (yes/no), recreational purposes (yes/no)	7
Obesity	- Open-ended question about weight and height §	7

Table footnotes:
* Included in the minimum dataset suggested by the Canadian Registry Working Group of the Strategy for Patient-Oriented Research (SPOR) Chronic Pain Network (CPN) (CPN, 2017)
† Core outcome domains recommended by the Initiative on Methods, Measurement, and Pain Assessment in Clinical Trials (IMMPACT) (Turk et al., 2003)
‡ Validated scales recommended by the Initiative on Methods, Measurement, and Pain Assessment in Clinical Trials (IMMPACT) (Dworkin et al., 2005)
§ Items from the Canadian Minimum dataset for chronic low back pain research (Lacasse et al., 2017)
|| Items from the Quebec Pain Registry (Choiniere et al., 2017)

References

Bem, S. L. (1974). The measurement of psychological androgyny. *Journal of consulting clinical psychology*, *42*(2), 155-162.

Bouhassira, D., Attal, N., Alchaar, H., Boureau, F., Brochet, B., Bruxelle, J., Cunin, G., Fermanian, J., Ginies, P., Grun-Overdyking, A., Jafari-Schluep, H., Lanteri-Minet, M., Laurent, B., Mick, G., Serrie, A., Valade, D., & Vicaut, E. (2005). Comparison of pain syndromes associated with nervous or somatic lesions and development of a new neuropathic pain diagnostic questionnaire (DN4). *Pain*, *114*(1-2), 29-36. https://doi.org/10.1016/j.pain.2004.12.010 (NOT IN FILE)

CADTH. (2018). *Access to and Availability of Non-Pharmacological Treatments for Chronic Non-Cancer Pain in Canada: An Environmental Scan*. Canadian Agency for Drugs and Technologies in Health (CADTH). https://www.cadth.ca/access-and-availability-non-pharmacological-treatments-chronic-non-cancer-pain-canada-environmental

Choiniere, M., Ware, M. A., Page, M. G., Lacasse, A., Lanctot, H., Beaudet, N., Boulanger, A., Bourgault, P., Cloutier, C., Coupal, L., De Koninck, Y., Dion, D., Dolbec, P., Germain, L., Martin, V., Sarret, P., Shir, Y., Taillefer, M. C., Tousignant, B., . . . Truchon, R. (2017). Development and Implementation of a Registry of Patients Attending Multidisciplinary Pain Treatment Clinics: The Quebec Pain Registry. *Pain Res Manag*, *2017*, 8123812. https://doi.org/10.1155/2017/8123812

Cleeland, C. S. (2009). *The Brief Pain Inventory User Guide*. The University of Texas MD Anderson Cancer Center. https://www.mdanderson.org/content/dam/mdanderson/documents/Departments-and-Divisions/Symptom-Research/BPI_UserGuide.pdf

CPN. (2017). *Chronic Pain Network Annual Report 2016/2017*. https://cpn.mcmaster.ca/docs/default-source/annual-reports/2017-annual-report-en.pdf?sfvrsn=2d511708_4

Dworkin, R. H., Turk, D. C., Farrar, J. T., Haythornthwaite, J. A., Jensen, M. P., Katz, N. P., Kerns, R. D., Stucki, G., Allen, R. R., Bellamy, N., Carr, D. B., Chandler, J.,

Cowan, P., Dionne, R., Galer, B. S., Hertz, S., Jadad, A. R., Kramer, L. D., Manning, D. C., . . . Witter, J. (2005). Core outcome measures for chronic pain clinical trials: IMMPACT recommendations. *Pain*, *113*(1-2), 9-19. http://ezproxy.usherbrooke.ca/login?url=http://search.ebscohost.com/login.aspx?direct=true&db=mnh&AN=15621359&site=ehost-live

Fontayne, P., Sarrazin, P., & Famose, J.-P. (2000). The Bem Sex-Role inventory: Validation of a short version for French teenagers. *European Review of Applied Psychology/Revue Européenne de Psychologie Appliquée*, *50*(4), 405-416.

Kroenke, K., Spitzer, R. L., Williams, J. B., & Lowe, B. (2009). An ultra-brief screening scale for anxiety and depression: the PHQ-4 [Evaluation Studies
Research Support, Non-U.S. Gov't]. *Psychosomatics*, *50*(6), 613-621. https://doi.org/10.1176/appi.psy.50.6.613

Lacasse, A., Roy, J. S., Parent, A. J., Noushi, N., Odenigbo, C., Page, G., Beaudet, N., Choiniere, M., Stone, L. S., & Ware, M. A. (2017). The Canadian minimum dataset for chronic low back pain research: a cross-cultural adaptation of the National Institutes of Health Task Force Research Standards. *CMAJ Open*, *5*(1), E237-E248. https://doi.org/10.9778/cmajo.20160117

Maruish, M. E. (2012). *User's manual for the SF-12v2 Health Survey* (3rd ed.). QualityMetric Incorporated.

Timmerman, H., Steegers, M. A. H., Huygen, F., Goeman, J. J., van Dasselaar, N. T., Schenkels, M. J., Wilder-Smith, O. H. G., Wolff, A. P., & Vissers, K. C. P. (2017). Investigating the validity of the DN4 in a consecutive population of patients with chronic pain. *PLoS One*, *12*(11), e0187961. https://doi.org/10.1371/journal.pone.0187961

Turk, D. C., Dworkin, R. H., Allen, R. R., Bellamy, N., Brandenburg, N., Carr, D. B., Cleeland, C., Dionne, R., Farrar, J. T., Galer, B. S., Hewitt, D. J., Jadad, A. R., Katz, N. P., Kramer, L. D., Manning, D. C., McCormick, C. G., McDermott, M. P., McGrath, P., Quessy, S., . . . Witter, J. (2003). Core outcome domains for chronic pain clinical trials: IMMPACT recommendations. *Pain*, *106*(3), 337-345. http://ezproxy.usherbrooke.ca/login?url=http://search.ebscohost.com/login.aspx?direct=true&db=mnh&AN=14659516&site=ehost-live

LABORATOIRE DE RECHERCHE
EN ÉPIDÉMIOLOGIE DE LA
DOULEUR CHRONIQUE

Mieux comprendre la douleur chronique et son traitement

Mot d'introduction et consentement

Projet de recherche

Mieux comprendre la douleur chronique et son traitement

Chercheure principale : Anaïs Lacasse, Ph.D., chercheure au Département des sciences de la santé de l'Université du Québec en Abitibi-Témiscamingue (UQAT)

Équipe de recherche : Véronique Gagnon, Inf., étudiante à la maîtrise recherche en sciences de la santé à l'UQAT ; Gabrielle Pagé, Ph.D., chercheure au Centre de recherche du Centre hospitalier de l'Université de Montréal ; Lucie Blais, Ph.D., chercheure à la Faculté de pharmacie de l'Université de Montréal ; Lyne Guénette, Ph.D., chercheure à la Faculté de pharmacie de l'Université Laval

Le présent projet de recherche vise à mieux comprendre la douleur chronique et son traitement. Vous êtes donc invité(e) à participer à cette étude menée par la professeure Anaïs Lacasse et son équipe. Ce projet de recherche a été financé par le Réseau québécois de recherche sur les médicaments (RQRM). Il a aussi été approuvé par le Comité d'éthique de la recherche de l'Université du Québec en Abitibi-Témiscamingue.

Pour être admissible, vous devez : 1) souffrir de douleur de façon constante ou occasionnelle depuis plus de 3 mois (peu importe si cette douleur est légère ou sévère et peu importe la cause), 2) être âgé(e) de 18 ans ou plus, 3) habiter au Québec, 4) être capable de répondre à un questionnaire en français.

Votre participation à ce projet de recherche consiste à consacrer environ 20 minutes de votre temps pour remplir notre questionnaire en ligne. Pendant que vous remplissez le questionnaire, vous pouvez prendre une pause, mais prière de laisser votre navigateur web ouvert afin de ne pas perdre les réponses déjà entrées.

Vous n'avez pas d'avantage immédiat relié à votre participation à cette étude et aucune indemnité compensatoire ne vous sera accordée. Toutefois, nos résultats contribueront à mieux comprendre l'usage, les bénéfices et les risques des traitements pour soulager la douleur chronique en contexte réel de pratique clinique. Le seul inconvénient découlant de votre participation, s'il en est, est le temps que vous consacrerez à remplir notre questionnaire en ligne.

Tous les renseignements obtenus sur vous dans le cadre de ce projet de recherche demeureront confidentiels. Pour éviter tout risque lié à la sécurité de vos données, prière d'éviter de répondre à ce questionnaire sur un réseau sans fil (WI-FI) public (ex. dans un café, aéroport, bibliothèque, etc.). Si vous travaillez sur un ordinateur partagé, il est aussi suggéré d'effacer votre historique de navigation.

Notre équipe de recherche affirme ne pas être en conflit d'intérêts réel, éventuel ou apparent avec le présent projet et ne pas viser aucune commercialisation des résultats.

Nous tenons à vous assurer que votre participation à cette étude est volontaire et que vous pouvez, en tout temps, sauter certaines questions ou arrêter de remplir notre questionnaire en ligne. En acceptant de participer à cette étude, vous ne renoncez à aucun de vos droits ni ne libérez les chercheurs ou le commanditaire de leurs obligations légales et professionnelles à votre égard. Si vous en faites la demande à la fin du questionnaire, il vous sera possible de recevoir un résumé des résultats par courriel.

Toute question concernant le projet pourra être adressée à Professeure Anaïs Lacasse : Douleur@uqat.ca / 1 877 870-8728, poste 2722

Pour tout renseignement supplémentaire concernant vos droits, vous pouvez vous adresser au :
Comité d'éthique de la recherche avec des êtres humains de l'Université du Québec en Abitibi-Témiscamingue
Vice-rectorat à l'enseignement et à la recherche
445, boulevard de l'Université, bureau B-309
Rouyn-Noranda (Québec) J9X 5E4
1 877 870-8728, poste 2252
cer@uqat.ca

*** J'accepte de participer au sondage et je confirme être âgé(e) de 18 ans et plus et habiter au Québec**

- ◯ Oui
- ◯ Non

Veuillez conserver un exemplaire de cette page pour vos dossiers

LABORATOIRE DE RECHERCHE EN ÉPIDÉMIOLOGIE DE LA DOULEUR CHRONIQUE

Mieux comprendre la douleur chronique et son traitement

Merci quand même pour votre intérêt!

Merci quand même pour votre intérêt!
Afin de savoir à quel point nos participants sont représentatifs de l'ensemble des personnes ayant entendu parler de notre sondage, nous vous invitons à répondre aux 3 questions suivantes :

Âge (18-120) :

Sexe :

○ Femme ○ Homme ○ Inconnu ○ Indéterminé

Raison(s) du refus?

LABORATOIRE DE RECHERCHE EN ÉPIDÉMIOLOGIE DE LA DOULEUR CHRONIQUE

Mieux comprendre la douleur chronique et son traitement

Caractéristiques de la douleur

*** Dans quelle(s) région(s) du corps ressentez-vous de la douleur?**

(cochez toutes les cases applicables)

- ☐ Douleurs généralisées dans tout le corps (ou presque)
- ☐ Tête
- ☐ Visage
- ☐ Cou
- ☐ Épaule(s)
- ☐ Bras
- ☐ Coude(s)
- ☐ Poignet(s)
- ☐ Main(s)
- ☐ Haut du dos
- ☐ Bas du dos
- ☐ Autre (veuillez préciser)
- ☐ Poitrine
- ☐ Abdomen / Estomac
- ☐ Hanche(s)
- ☐ Fesse(s)
- ☐ Région anale
- ☐ Région génitale
- ☐ Jambe(s)
- ☐ Genou(x)
- ☐ Cheville(s)
- ☐ Pied(s)

Si vous souffrez de douleur dans plus d'une région du corps, veuillez répondre à toutes les questions suivantes en tenant compte de l'endroit qui fait le plus mal

*** Indiquez les circonstances qui, selon vous, ont mené à l'apparition de votre douleur (cochez plus d'une réponse s'il y a lieu)**

- ☐ Accident au travail
- ☐ Accident à la maison
- ☐ Accident avec véhicule motorisé
- ☐ Accident de sport
- ☐ À la suite d’une chirurgie
- ☐ À la suite d’un événement stressant
- ☐ Postures ou mouvements répétitifs
- ☐ Durant ou à la suite d’un cancer
- ☐ Durant ou à la suite d’une maladie (autre que le cancer)
- ☐ Maladie dégénérative liée à l’âge (ex. arthrose)
- ☐ Maladie inflammatoire ou auto-immune (ex. arthrite rhumatoïde, lupus)
- ☐ Aucun événement précis
- ☐ Je ne sais pas
- ☐ Autre raison ou événement (veuillez préciser)

Est-ce que votre douleur est :

- ○ Présente continuellement
- ○ Présente occasionnellement

Depuis combien de temps ressentez-vous votre douleur?

En jours :

OU

En mois :

OU

En années :

Intensité de la douleur

	0 (AUCUNE DOULEUR)	1	2	3	4	5	6	7	8	9	10 (LA PIRE DOULEUR POSSIBLE)
Veuillez choisir sur l'échelle suivante le chiffre qui décrit le mieux <u>la pire douleur</u> que vous avez ressentie au cours des 7 derniers jours	○	○	○	○	○	○	○	○	○	○	○
Veuillez choisir sur l'échelle suivante le chiffre qui décrit le mieux la douleur que vous avez ressentie <u>en moyenne ou en général</u> au cours des 7 derniers jours	○	○	○	○	○	○	○	○	○	○	○

Êtes-vous en accord avec l'énoncé suivant :
Je considère que ma douleur est épouvantable et j'ai l'impression que cela ne s'améliorera jamais.

○ D'accord

○ Pas d'accord

LABORATOIRE DE RECHERCHE EN ÉPIDÉMIOLOGIE DE LA DOULEUR CHRONIQUE

Mieux comprendre la douleur chronique et son traitement

Composante neuropathique de la douleur (questionnaire DN4)

Si vous souffrez de douleur dans plus d'une région du corps, veuillez répondre à toutes les questions suivantes en tenant compte de l'endroit qui fait le plus mal

La douleur présente-t-elle une ou plusieurs des caractéristiques suivantes ?

	Oui	Non
Brûlure	○	○
Sensation de froid douloureux	○	○
Décharges électriques	○	○

La douleur est-elle associée dans la même région à un ou plusieurs des symptômes suivants?

	Oui	Non
Fourmillements	○	○
Picotements	○	○
Engourdissement	○	○
Démangeaisons	○	○

LABORATOIRE DE RECHERCHE EN ÉPIDÉMIOLOGIE DE LA DOULEUR CHRONIQUE

Mieux comprendre la douleur chronique et son traitement

Interférence de la douleur

Veuillez choisir sur les échelles suivantes le chiffre qui décrit le mieux comment, <u>au cours des 7 derniers jours</u>, la douleur a gêné votre :

	0 (NE GÊNE PAS)	1	2	3	4	5	6	7	8	9	10 (GÊNE COMPLÈTEMENT)
Activité générale	○	○	○	○	○	○	○	○	○	○	○
Humeur	○	○	○	○	○	○	○	○	○	○	○
Capacité à marcher	○	○	○	○	○	○	○	○	○	○	○
Travail habituel (y compris le travail à l'extérieur de la maison et les travaux domestiques)	○	○	○	○	○	○	○	○	○	○	○
Relation avec les autres	○	○	○	○	○	○	○	○	○	○	○
Sommeil	○	○	○	○	○	○	○	○	○	○	○
Goût de vivre	○	○	○	○	○	○	○	○	○	○	○

N.B. si vous n'avez ressenti aucune douleur au cours des 7 derniers jours, veuillez choisir " 0 " sur les échelles ci-dessus

UQAT

LABORATOIRE DE RECHERCHE
EN ÉPIDÉMIOLOGIE DE LA
DOULEUR CHRONIQUE

Mieux comprendre la douleur chronique et son traitement

Jumelage confidentiel de données personnelles

Nous aimerions en connaître davantage sur votre utilisation de médicaments prescrits et de soins de santé (ex. consultations dans les hôpitaux, cliniques et bureaux de médecins). Pour ce faire, nous vous demandons la permission de jumeler les données recueillies dans ce questionnaire aux données de la **Régie de l'assurance maladie du Québec (RAMQ)**. Ceci nous permettra de recevoir les informations au sujet des médicaments et des services de santé que vous avez utilisés dans les 5 dernières années et que vous utiliserez dans la prochaine année.

Ces renseignements jumelés demeureront strictement confidentiels et ne seront utilisés qu'à des fins de recherche. **Notez que vous pouvez refuser, mais répondre au reste du questionnaire.** Un refus ne modifiera en rien la qualité ou la quantité des soins ou services de santé que vous recevez ou auxquels vous avez droit.

Si vous acceptez, une personne désignée au sein de l'équipe de recherche transmettra votre prénom, nom de famille, date de naissance, sexe, numéro de carte d'assurance maladie et informations sur votre assurance à la RAMQ (après avoir obtenu l'approbation de la Comission d'accès à l'information du Québec).

Tous les transferts d'information seront effectués par courrier recommandé et fichiers informatiques sécurisés. L'équipe de recherche procédera ensuite au jumelage des bases de données. Afin de préserver votre identité et la confidentialité de vos renseignements personnels, toutes les informations permettant de vous identifier seront par la suite effacées de la banque de données et vous serez identifié(e) que par un numéro de code. Il nous serait donc impossible de détruire les données d'un participant qui en ferait la demande.

Est-ce que vous nous donnez la permission d'effectuer ce jumelage?

○ Oui ○ Non

<u>Sinon</u>, prière de nous indiquer pour quelle(s) raison(s) et passer à la page suivante

<u>Si oui</u>, prière de compléter les sections suivantes :

Prénom :

Nom de famille :

Numéro d'assurance maladie tel qu'il apparaît sur votre carte d'assurance maladie du Québec **(sans espaces)** :

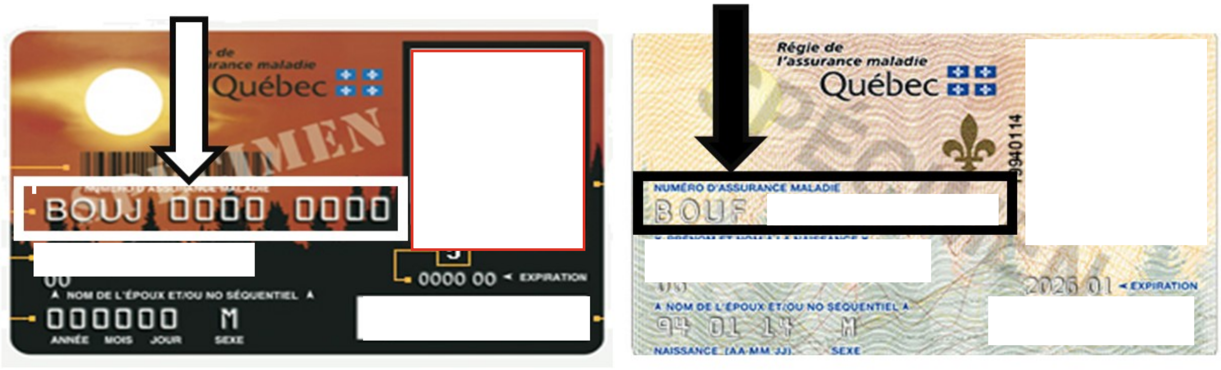

Disponible en libre accès via : https://www.ramq.gouv.qc.ca/fr/citoyens/assurance-maladie/utilisation-carte

Date de naissance

Date de naissance

JJ/MM/AAAA

Pour vos médicaments sur ordonnance (médicaments prescrits), avez-vous eu à un moment ou à un autre une assurance médicaments collective ou privée au cours de la dernière année?

- ◯ Oui
- ◯ Non
- ◯ Je ne sais pas

L’assurance médicament du gouvernement (RAMQ) couvre seulement une portion de la population québécoise et il nous est impossible d’avoir accès via la RAMQ aux données liées aux médicaments achetés par les personnes qui ont une assurance médicaments collective ou privée. Un registre permettant cet accès appelé reMed est cependant maintenu par professeure Lucie Blais, collaboratrice au présent projet et chercheure à la Faculté de Pharmacie de l’Université de Montréal. Cet accès nécessite cependant un consentement séparé et une inscription au registre reMed. Accepteriez-vous que notre équipe communique avec vous par courriel ou par la poste pour mettre en place cet accès?

◯ Oui

◯ Non

<u>Sinon</u>, passez à la page suivante.

<u>Si oui</u>, prière d'inscrire votre adresse courriel dans la case ci-dessous :

[ ]

Si vous n’avez pas d’adresse courriel, vous pouvez toujours nous laisser votre adresse postale :

Nom [ ]

Adresse [ ]

Adresse 2 [ ]

Ville/Localité [ ]

Province [ ]

Code postal [ ]

Pays [ ]

UQAT
LABORATOIRE DE RECHERCHE
EN ÉPIDÉMIOLOGIE DE LA
DOULEUR CHRONIQUE

Mieux comprendre la douleur chronique et son traitement

Traitement de la douleur

Pour le traitement de votre douleur, utilisez-vous actuellement des médicaments prescrits (qui nécessitent une ordonnance de la part d'un médecin, d'un pharmacien ou d'une infirmière praticienne)?

- ◯ Oui
- ◯ Non

Pour le traitement de votre douleur, utilisez-vous actuellement des médicaments en vente libre (qui ne nécessitent pas d'ordonnance, par exemple: Robax®, Tylenol®, ou Advil®)?

- ◯ Oui
- ◯ Non

Ressentez-vous l'un ou l'autre des effets secondaires suivants à cause de votre traitement actuel contre la douleur?

	Aucun	Léger	Modéré	Sévère
Vertige / étourdissement	○	○	○	○
Somnolence	○	○	○	○
Confusion	○	○	○	○
Nausée	○	○	○	○
Vomissement	○	○	○	○
Perte de mémoire	○	○	○	○
Bouche sèche	○	○	○	○
Démangeaison	○	○	○	○
Inconfort abdominal	○	○	○	○
Constipation	○	○	○	○
Ralentissement du débit urinaire	○	○	○	○
Fatigue	○	○	○	○
Insomnie	○	○	○	○
Enflure	○	○	○	○
Gain de poids	○	○	○	○
Vision brouillée	○	○	○	○
Baisse de libido	○	○	○	○
Hallucinations	○	○	○	○
Cauchemars	○	○	○	○

Autre (veuillez préciser)

[ ]

Mis à part des médicaments, utilisez-vous actuellement d'autres types de traitements pour votre douleur?

○ Oui

○ Non

Si oui, lesquels (cochez toutes les réponses qui s'appliquent)?

- ☐ Acupuncture /Acupression
- ☐ Aquathérapie/Hydrothérapie (ex. bain, aqua forme)
- ☐ Aromathérapie (ex. extraits de plantes, huiles essentielles)
- ☐ Biofeedback
- ☐ Chaud et/ou froid
- ☐ Chiropratique
- ☐ Ergothérapie
- ☐ Exercice/activité physique
- ☐ Groupes de soutien/d'entraide (qui regroupe seulement des patients)
- ☐ Hypnose
- ☐ Implant d'un neurostimulateur
- ☐ Autre (veuillez préciser)
- ☐ Injections (ex. bloc ou autres techniques)
- ☐ Intervention de groupe (ex. école du dos)
- ☐ Massothérapie / Massage
- ☐ Méditation
- ☐ Musicothérapie (thérapie par la musique)
- ☐ Ostéopathie
- ☐ Physiothérapie
- ☐ Produits homéopathiques
- ☐ Produits naturels
- ☐ Psychothérapie
- ☐ Réalité virtuelle ou augmentée
- ☐ Réflexologie
- ☐ Reiki
- ☐ Stimulation nerveuse électrique transcutanée (TENS)
- ☐ Taï Chi
- ☐ Taping (bandes élastiques)
- ☐ Techniques de relaxation/respiration
- ☐ Thérapie cognitivo comportementale
- ☐ Thérapie comportementale
- ☐ Yoga
- ☐ Zoothérapie (thérapie grâce aux animaux)

[ ]

Dans l'ensemble, quel soulagement les traitements ou médicaments utilisés <u>actuellement</u> pour votre douleur vous apportent-ils?

	0 % (AUCUN SOULAGEMENT)	10 %	20 %	30 %	40 %	50 %	60 %	70 %	80 %	90 %	100 % (SOULAGEMENT COMPLET)
Choix de réponses :	○	○	○	○	○	○	○	○	○	○	○

De tous vos traitements utilisés <u>actuellement</u> (incluant les médicaments, les approches non pharmacologiques, les interventions, etc.), qu'est-ce qui vous apparaît le plus efficace?

[ ]

Avez-vous accès à un <u>professionnel de la santé</u> en qui vous avez confiance pour le traitement de votre douleur (ex. médecin, infirmière, pharmacien, physiothérapeute, psychologue, etc.)?

○ Oui

○ Non

LABORATOIRE DE RECHERCHE EN ÉPIDÉMIOLOGIE DE LA DOULEUR CHRONIQUE

Mieux comprendre la douleur chronique et son traitement

Profil sociodémographique

Âge (18-120) :

[ ]

○ Femme

○ Homme

○ Inconnu

○ Indéterminé

Êtes-vous autochtone, c'est-à-dire, Première Nation, Métis ou Inuk (Inuit)?

☐ Non, je ne suis pas autochtone

☐ Oui, Première Nation - Indien(ne) de l'Amérique du Nord

☐ Oui, Métis

☐ Oui, Inuk (Inuit)

Quel est votre pays de naissance?

○ Canada

○ Autre (veuillez préciser)

[ ]

Êtes-vous?

- ☐ Blanc/Blanche
- ☐ Sud-Asiatique - ex. Indien(ne) de l’Inde, Pakistanais(e), Sri-Lankais(e)
- ☐ Chinois(e)
- ☐ Noir(e)
- ☐ Philippin(e)
- ☐ Latino-Américain(e)
- ☐ Arabe
- ☐ Asiatique du Sud-Est - ex. Vietnamien(ne), Cambodgien(ne), Malaisien(ne), Laotien(ne)
- ☐ Asiatique occidental(e) - ex. Iranien(ne), Afghan(e)
- ☐ Coréen(ne)
- ☐ Japonais(e)
- ☐ Autre :

(veuillez préciser)

Quel statut d’emploi représente le mieux votre situation?

- ◯ Travaille présentement à temps plein
- ◯ Travaille présentement à temps partiel
- ◯ À la recherche d’emploi / sans emploi
- ◯ Congé de maladie
- ◯ Congé de maternité
- ◯ Invalidité temporaire ou permanente en raison de votre douleur
- ◯ Invalidité pour d’autres raisons que la douleur
- ◯ Étudiant
- ◯ Mise à pied temporaire (chômage planifié)
- ◯ Retraité(e)
- ◯ Au foyer
- ◯ Inconnu
- ◯ Autre :
- ◯ (veuillez préciser)

Êtes-vous actuellement impliqué(e) dans un litige pour obtenir des prestations d’invalidité ?

Par exemple avec l’employeur, un assureur, la Commission des normes, de l'équité, de la santé et de la sécurité du travail (CNESST), la Société de l’Assurance automobile du Québec (SAAQ) ou l’Indemnisation des victimes d’actes criminels (IVAC)

- ◯ Oui
- ◯ Non
- ◯ Incertain(e)

Niveau de scolarité (sélectionnez le plus haut niveau atteint) :

- ◯ Sans diplôme d’études secondaires
- ◯ Diplôme d'études secondaires ou l'équivalent
- ◯ Certificat ou diplôme d'apprenti inscrit, d'une école de métiers ou d'un centre de formation professionnelle? (ex. coiffure, cuisine, électricien, charpenterie)
- ◯ Certificat ou diplôme d'études collégiales, d'un CÉGEP ou d'un autre établissement non universitaire (ex. technique de comptabilité, technique de génie industriel, adjoint juridique, DEC préuniversitaire, etc.)
- ◯ Programme universitaire de 1er cycle (ex. baccalauréat, certificat)
- ◯ Programme universitaire de 2e cycle (ex. maîtrise, D.E.S.S., microprogramme de 2e cycle)
- ◯ Doctorat (ex. MD, PharmD, Ph.D., D.Psy., D.Ed.)

Dans quelle région du Québec habitez-vous présentement?

- ◯ Abitibi-Témiscamingue
- ◯ Bas-Saint-Laurent
- ◯ Capitale Nationale
- ◯ Centre-du-Québec
- ◯ Chaudière-Appalaches
- ◯ Côte-Nord
- ◯ Estrie
- ◯ Gaspésie-Îles-de-la-Madeleine
- ◯ Lanaudière
- ◯ Laurentides
- ◯ Laval
- ◯ Mauricie
- ◯ Montérégie
- ◯ Montréal
- ◯ Nord-du-Québec
- ◯ Outaouais
- ◯ Saguenay-Lac-Saint-Jean
- ◯ Autre (veuillez préciser)

Svp, indiquez à quel point chacune des caractéristiques ci-dessous vous représente :

	1 (jamais ou presque jamais vrai)	2	3	4	5	6	7 (presque toujours vrai)
J'ai confiance en moi	◯	◯	◯	◯	◯	◯	◯
J'aime rendre service	◯	◯	◯	◯	◯	◯	◯
Je suis sportif(ve)	◯	◯	◯	◯	◯	◯	◯

	1 (jamais ou presque jamais vrai)	2	3	4	5	6	7 (presque toujours vrai)
Je suis affectueux(se)	○	○	○	○	○	○	○
Je suis sûr(e) de moi	○	○	○	○	○	○	○
Je suis énergique	○	○	○	○	○	○	○
Je suis toujours prêt(e) à écouter les autres	○	○	○	○	○	○	○
J'ai des qualités de commandement	○	○	○	○	○	○	○
Je suis attentif(ve) aux besoins des autres	○	○	○	○	○	○	○
Je suis sensible aux peines et aux problèmes des autres	○	○	○	○	○	○	○
Je suis prêt(e) à consoler les gens	○	○	○	○	○	○	○
Je suis dominateur(trice)	○	○	○	○	○	○	○
Je suis chaleureux(se)	○	○	○	○	○	○	○
Je suis tendre	○	○	○	○	○	○	○
Je me comporte en chef	○	○	○	○	○	○	○
J'ai l'esprit de compétition	○	○	○	○	○	○	○
J'aime les enfants	○	○	○	○	○	○	○
Je suis doux(ce)	○	○	○	○	○	○	○

UQAT
LABORATOIRE DE RECHERCHE
EN ÉPIDÉMIOLOGIE DE LA
DOULEUR CHRONIQUE

Mieux comprendre la douleur chronique et son traitement

Informations générales sur votre santé

En général, diriez-vous que votre santé est :

- ◯ Excellente
- ◯ Très bonne
- ◯ Bonne
- ◯ Passable
- ◯ Mauvaise

Combien de médicaments différents utilisez-vous <u>actuellement</u> (qu'ils soient prescrits ou en vente libre ; qu'ils soient pour la douleur ou tout autre problème de santé)?

Au cours des 2 dernières semaines, à quelle fréquence avez-vous été dérangé(e) par les problèmes suivants?

	Jamais	Plusieurs jours	Plus de 7 jours	Presque tous les jours
Sentiment de nervosité, d’anxiété ou de tension	○	○	○	○
Incapable d’arrêter de vous inquiéter ou de contrôler vos inquiétudes	○	○	○	○
Peu d’intérêt ou de plaisir à faire des choses	○	○	○	○
Se sentir triste, déprimé ou désespéré	○	○	○	○

Les questions suivantes portent sur les activités que vous pourriez avoir à faire au cours d'une journée normale. Votre état de santé actuel vous limite-t-il dans ces activités? Si oui, dans quelle mesure?

	Mon état de santé me limite beaucoup	Mon état de santé me limite un peu	Mon état de santé ne me limite pas du tout
Dans les activités modérées comme déplacer une table, passer l'aspirateur, jouer aux quilles ou au golf	○	○	○
Pour monter plusieurs étages à pied	○	○	○

Au cours de la dernière année...

	Jamais	Rarement	Parfois	Souvent
Avez-vous consommé de l'alcool ou des drogues plus que vous ne l'auriez voulu?	○	○	○	○
Avez-vous déjà voulu ou ressenti le besoin de réduire votre consommation d'alcool ou de drogues?	○	○	○	○

Au cours de la dernière année, avez-vous utilisé du cannabis?

	Oui	Non
Pour la gestion de votre douleur	○	○
Pour la gestion d'autres problèmes de santé	○	○
À des fins récréatives (pour le plaisir)	○	○

Quel énoncé décrit le mieux vos habitudes par rapport à la cigarette?

- ○ Je n'ai jamais fumé
- ○ Je suis un fumeur
- ○ J'ai déjà fumé, mais je ne fume plus

Taille :

En pieds/pouces (ex. 5'6) : ____________________

OU

En centimètres (cm) : ____________________

Poids :

En livres (lbs) :

OU

En kilogrammes (kg) :

Comment avez-vous entendu parler de ce sondage?

- ◯ Facebook
- ◯ Twitter
- ◯ Instagram
- ◯ Courriel reçu de la part d'un ami, d'un membre de votre famille, d'un collègue de travail ou d'un professeur
- ◯ Courriel reçu de la part d'une association de patients (ex. Association québécoise de la douleur chronique, Association de la fibromyalgie, Société de l'arthrite)
- ◯ Site web de l'Université du Québec en Abitibi-Témiscamingue (UQAT)
- ◯ Site web d'une station de radio
- ◯ Entendu à la radio
- ◯ Journal en format papier
- ◯ Autre (veuillez préciser)

Le questionnaire sur la douleur et son traitement se termine ici

Aimeriez-vous recevoir un résumé des résultats à la fin du projet?

◯ Oui ◯ Non

Aimeriez-vous être recontacté(e) pour de futures études réalisées par notre groupe de recherche?

◯ Oui ◯ Non

Si vous avez répondu oui à l'une ou l'autre des 2 questions précédentes, svp nous laisser votre adresse courriel dans la case ci-dessous :

Si vous n’avez pas d’adresse courriel, vous pouvez toujours nous laisser votre adresse postale :

Nom

Adresse

Adresse 2

Ville/Localité

Province

Code postal

Pays

Merci beaucoup de votre participation!

Nous sommes très reconnaissants que vous ayez accepté de nous aider dans nos recherches!

UQAT

LABORATOIRE DE RECHERCHE EN ÉPIDÉMIOLOGIE DE LA DOULEUR CHRONIQUE

LABORATOIRE DE RECHERCHE EN ÉPIDÉMIOLOGIE DE LA DOULEUR CHRONIQUE

Please note that only the French questionnaire was used in this study. The English version is provided here to help in understanding the article.

Better understanding chronic pain and its treatment

Introduction and Consent Statement

Research project

Better understanding chronic pain and its treatment

Principal Investigator: Anaïs Lacasse, Ph.D., researcher in Health Sciences at the Université du Québec en Abitibi-Témiscamingue (UQAT)

Research team: Véronique Gagnon, nurse., Master's student in Health Sciences at UQAT ; Gabrielle Pagé, Ph.D., researcher at the Research Center of the Centre hospitalier de l'Université de Montréal (CRCHUM); Lucie Blais, Ph.D., Researcher at the Faculty of Pharmacy, University of Montreal; Lyne Guénette, Ph.D., Researcher at the Faculty of Pharmacy, Laval University

The goal of this research project is to better understand chronic pain and its treatment. You are therefore invited to participate in this study led by Professor Anaïs Lacasse and her team. This research project is funded by the Quebec Medication Research Network (Réseau québécois de recherche sur les médicaments – RQRM). It has also been approved by the Research Ethics Committee of the Université du Québec en Abitibi-Témiscamingue.

To be eligible, you must: 1) experience pain either constantly or occasionally for more than 3 months (regardless of whether the pain is mild or severe, and regardless of the cause), 2) be 18 years of age or older, 3) reside in the province of Quebec, 4) be able to complete a questionnaire in French.

Your participation in this research project involves spending approximately 20 minutes to complete our online questionnaire. While filling out the questionnaire, you may take a break at any time; however, please keep your web browser open so that your previously entered answers are not lost.

You will not receive any immediate benefit from participating in this study, and no financial compensation will be provided. However, the results will contribute to a better understanding of the use, benefits, and risks of treatments for chronic pain in real-world clinical practice. The only potential inconvenience related to your participation is the time required to complete our online questionnaire.

All information collected about you as part of this research project will remain confidential. To minimize any risk related to data security, please avoid completing this questionnaire on a public wireless (Wi-Fi) network (e.g., in a café, airport, library, etc.). If you are using a shared computer, it is also recommended that you clear your browsing history afterward.

Our research team declares that it has no actual, potential, or apparent conflict of interest related to this project and that it does not intend to commercialize the results.

We want to assure you that your participation in this study is voluntary, and that you may skip any question or stop completing the online questionnaire at any time. By agreeing to participate in this study, you do not waive any of your rights, nor do you release the researchers or the sponsor from their legal and professional responsibilities toward you. If you request it at the end of the questionnaire, you will be able to receive a summary of the results by email.

Any questions regarding the project can be addressed to Professor Anaïs Lacasse at :
Douleur@uqat.ca / 1 877 870-8728, extension 2722

For any additional information regarding your rights, you may contact:
The Research Ethics Committee for Research Involving Human Subjects at the Université du Québec en Abitibi-Témiscamingue
Vice-Rectorate for Teaching and Research
445, boulevard de l'Université, office B-309
Rouyn-Noranda (Québec) J9X 5E4
1 877 870-8728, extension 2252
cer@uqat.ca

*** I agree to participate in the survey and confirm that I am 18 years of age or older and reside in Quebec.**

○ Yes

○ No

Please keep a copy of this page for your records

UQAT
LABORATOIRE DE RECHERCHE
EN ÉPIDÉMIOLOGIE DE LA
DOULEUR CHRONIQUE

Better understanding chronic pain and its treatment

Thank you anyway for your interest!

Thank you anyway for your interest!
To understand how representative our participants are of the overall population aware of our survey, we invite you to answer the following 3 questions:

Age (18-120) :

[ ]

Sex :

○ Women ○ Men ○ Unknown ○ Undetermined

Reason(s) for refusal?

[ ]

UQAT
LABORATOIRE DE RECHERCHE
EN ÉPIDÉMIOLOGIE DE LA
DOULEUR CHRONIQUE

Better understanding chronic pain and its treatment

Pain characteristics

*** In which areas of your body do you experience pain? (Check all that apply)**

- ☐ Widespread pain throughout the body (or almost)
- ☐ Head
- ☐ Face
- ☐ Neck
- ☐ Shoulder(s)
- ☐ Arm(s)
- ☐ Elbow(s)
- ☐ Wrist(s)
- ☐ Hand(s)
- ☐ Upper back
- ☐ Lower back
- ☐ Other (please specify):
- ☐ Chest
- ☐ Abdomen/Stomach
- ☐ Hip(s)
- ☐ Buttock(s)
- ☐ Anal area
- ☐ Genital area
- ☐ Leg(s)
- ☐ Knee(s)
- ☐ Ankle(s)
- ☐ Foot/feet

If you experience pain in more than one body region, please answer all of the following questions based on the area where the pain is the most severe

*** Indicate the circumstances that you believe led to the onset of your pain (or the different types of pain you have) (Check all that apply):**

- [ ] Work-related accident
- [ ] Accident at home
- [ ] Motor vehicle accident
- [ ] Sport-related accident
- [ ] Following surgery
- [ ] Following a stressful event
- [ ] Repetitive postures or movements
- [ ] During or following cancer
- [ ] During or following another illness (not cancer)
- [ ] Age-related degenerative disease (e.g., osteoarthritis)
- [ ] Inflammatory or autoimmune disease (e.g., rheumatoid arthritis, lupus)
- [ ] No specific event
- [ ] I don't kow
- [ ] Other reason or event (please specify):

Is your pain :

- ○ Present continuously
- ○ Present occasionally

How long have you been experiencing your pain?

In days :

OR

In months :

OR

In years :

Pain intensity

	0 (NO PAIN)	1	2	3	4	5	6	7	8	9	10 (WORST POSSIBLE PAIN)
Please select on the next scales, the one number that best describes your pain at its worst in the past 7 days	◯	◯	◯	◯	◯	◯	◯	◯	◯	◯	◯
Please select on the next scales, the one number that best describes your pain on the average or at its usual level in the past 7 days	◯	◯	◯	◯	◯	◯	◯	◯	◯	◯	◯

Do you agree with the following statement:
It’s terrible and I think it’s never going to get any better

◯ Agree

◯ Disagree

UQAT
LABORATOIRE DE RECHERCHE EN ÉPIDÉMIOLOGIE DE LA DOULEUR CHRONIQUE

Better understanding chronic pain and its treatment

Neuropathic Component of Pain (DN4 Questionnaire)

If you experience pain in more than one area of your body, please answer all of the following questions based on the area where the pain is most intense.

Does the pain have one or more of the following characteristics?

	Yes	No
Burning	○	○
Painful cold	○	○
Electric shocks	○	○

Is the pain associated with one or more of the following symptoms in the same area?

	Yes	No
Tingling	○	○
Pins and needles	○	○
Numbness	○	○
Itching	○	○

UQAT
LABORATOIRE DE RECHERCHE
EN ÉPIDÉMIOLOGIE DE LA
DOULEUR CHRONIQUE

Better understanding chronic pain and its treatment

Pain interference

Please select on the next scale, the one number that best describes how, during the past 7 days, pain has interfered with:

	0 (DOES NOT INTERFERE)	1	2	3	4	5	6	7	8	9	10 (COMPLETELY INTERFERES)
General activity	○	○	○	○	○	○	○	○	○	○	○
Mood	○	○	○	○	○	○	○	○	○	○	○
Walking ability	○	○	○	○	○	○	○	○	○	○	○
Normal work (includes both work outside the home and housework)	○	○	○	○	○	○	○	○	○	○	○
Relations with other people	○	○	○	○	○	○	○	○	○	○	○
Sleep	○	○	○	○	○	○	○	○	○	○	○
Enjoyment of life	○	○	○	○	○	○	○	○	○	○	○

Note: If you have not experienced any pain in the past 7 days, please select "0" on the above scales.

UQAT
LABORATOIRE DE RECHERCHE EN ÉPIDÉMIOLOGIE DE LA DOULEUR CHRONIQUE

Better understanding chronic pain and its treatment

Confidential Linking of Personal Data

We would like to learn more about your use of prescription medications and public healthcare services (e.g., hospital consultations, clinics, and physicians' offices). To do this, we are asking for your permission to link the responses you provided in this questionnaire with data from the ***Régie de l'assurance maladie du Québec (RAMQ)*.** This will allow us to access information about the medications and healthcare services you have used in the past five years and those you will use over the next year.

These linked data will remain strictly confidential and will be used only for research purposes. **Please note that you may refuse, but still complete the rest of the questionnaire**. Refusal will in no way affect the quality or quantity of health care or services you receive or are entitled to.

If you agree, a designated person within the research team will send your first name, last name, date of birth, sex, health insurance card number, and insurance information to the RAMQ (after obtaining approval from the *Commission d'accès à l'information (CAI) du Québec*).

All information transfers will be carried out by registered mail and secure electronic files. The research team will then perform the data linking. To protect your identity and the confidentiality of your personal information, all identifying details will be deleted from the database afterward, and you will be identified only by a code number. Therefore, it would be impossible for us to delete the data of a participant who requests it.

Do you give us permission to carry out this data linking?

◯ Yes ◯ No

Otherwise, please indicate the reason(s) and proceed to the next page.

If yes, please complete the following sections:

First name :

Last name :

Health insurance number as it appears on your Québec Health Insurance Card, as shown in the example below
(please enter the 12 caracters without spaces):

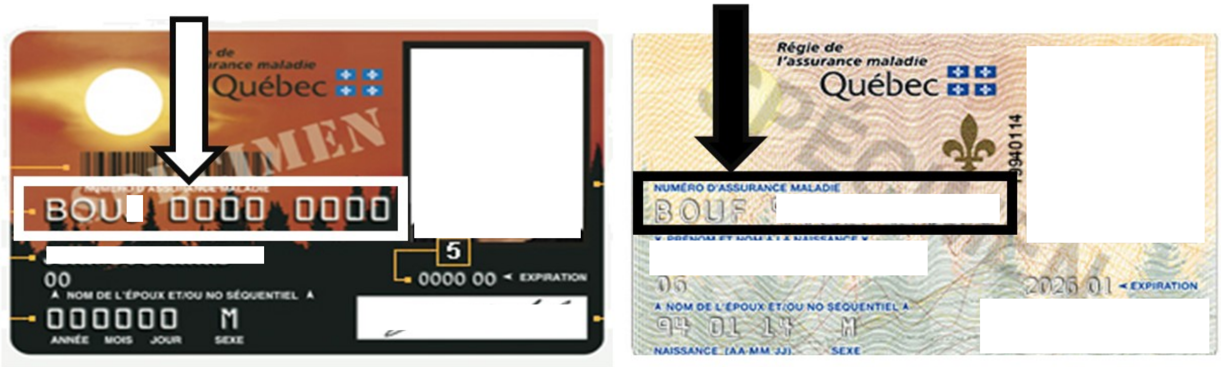

Publicly available online at : https://www.ramq.gouv.qc.ca/en/citizens/health-insurance/using-card

Date of birth

Date of birth

JJ/MM/AAAA

For your prescription medications, have you had group or private drug insurance at any time during the past year?

- ◯ Yes
- ◯ No
- ◯ I don't know

The government drug insurance plan (RAMQ) covers only a portion of the Quebec population, and it is not possible for us to access medication data through the RAMQ for individuals who have group or private drug insurance. However, a registry that allows such access, called reMed, is maintained by Professor Lucie Blais, a collaborator on this project and a researcher at the Faculty of Pharmacy at the Université de Montréal. Access to this registry requires a separate consent and registration with the reMed registry. Would you agree to let our team contact you by email or by mail to set up this access?

- ◯ Yes
- ◯ No

Otherwise, please proceed to the next page

<u>If yes</u>, please enter your email address in the box below:

[ ]

If you do not have an email address, you may still provide your mailing address:

Name [ ]

Adress [ ]

Adresse Line 2 [ ]

City/Town [ ]

Province [ ]

Postal Code [ ]

Country [ ]

LABORATOIRE DE RECHERCHE
EN ÉPIDÉMIOLOGIE DE LA
DOULEUR CHRONIQUE

Better understanding chronic pain and its treatment

Treatment of pain

For the treatment of your pain, are you currently using prescribed medications, meaning those that require a prescription from a doctor, pharmacist, or nurse practitioner?

◯ Yes

◯ No

For the treatment of your pain, are you currently using over-the-counter medications (those that do not require a prescription, such as: Robax®, Tylenol®, Advil®)?

◯ Yes

◯ No

Are you experiencing any of the following side effects from your current treatment for pain?

	None	Mild	Moderate	Severe
Lightheadedness /dizziness	◯	◯	◯	◯
Drowsiness	◯	◯	◯	◯
Confusion	◯	◯	◯	◯
Nausea	◯	◯	◯	◯
Vomiting	◯	◯	◯	◯
Impaired memory	◯	◯	◯	◯
Dry mouth	◯	◯	◯	◯
Itching	◯	◯	◯	◯
Abdominal discomfort	◯	◯	◯	◯
Constipation	◯	◯	◯	◯
Slowing of the urine stream	◯	◯	◯	◯
Fatigue	◯	◯	◯	◯
Insomnia	◯	◯	◯	◯
Swelling	◯	◯	◯	◯
Weight gain	◯	◯	◯	◯
Visual blurring	◯	◯	◯	◯
Decreased sex drive	◯	◯	◯	◯
Hallucinations	◯	◯	◯	◯
Nightmares	◯	◯	◯	◯

Other (please specify):

Apart from medication, do you currently use any other types of treatment for your pain?

◯ Yes

◯ No

If yes, which ones (check all that apply)?

- ☐ Acupuncture/Acupressure
- ☐ Aquatictherapy/Hydrotherapy (e.g., baths, aqua fitness)
- ☐ Aromatherapy (e.g., plant extracts, essential oils)
- ☐ Biofeedback
- ☐ Hot-Cold treatmens
- ☐ Chiropractic care
- ☐ Ergotherapy
- ☐ Exercises/physical activity
- ☐ Support groups (comprised exclusively of patients)
- ☐ Hypnosis
- ☐ Neurostimulator implantation
- ☐ Other (please specify):
- ☐ Injections (e.g., nerve blocks or other techniques)
- ☐ Group intervention (e.g., back school)
- ☐ Massage therapy/Massage
- ☐ Meditation
- ☐ Music therapy (therapy by music)
- ☐ Osteopathiy
- ☐ Physiotherapy
- ☐ Homeopathic products
- ☐ Naturals products
- ☐ Psychotherapy
- ☐ Virtual/augmented reality
- ☐ Reflexology
- ☐ Reiki
- ☐ Transcutaneous electrical nerve stimulation (TENS)
- ☐ Tai Chi
- ☐ Tapping (elastic bands)
- ☐ Relaxation/respiration
- ☐ Cognitive behavioural therapy
- ☐ Behavioural therapy
- ☐ Yoga
- ☐ Zootherapy (animal-assisted therapy)

[ ]

Overall, how much relief do you get from the treatments or medications currently used in your treatment?

	0 % (NO RELIEF)	10 %	20 %	30 %	40 %	50 %	60 %	70 %	80 %	90 %	100 % (COMPLETE RELIEF)
Response options:	○	○	○	○	○	○	○	○	○	○	○

Of all the treatments you are currently using (including medications, non-pharmacological approaches, interventions, etc.), which do you find the most effective?

[ ]

Do you have access to a healthcare professional you trust for the treatment of your pain (e.g., doctor, nurse, pharmacist, physiotherapist, psychologist, etc.)?

○ Yes

○ No

LABORATOIRE DE RECHERCHE
EN ÉPIDÉMIOLOGIE DE LA
DOULEUR CHRONIQUE

Better understanding chronic pain and its treatment

Socio-demographic profile

Age (18-120) :

[ ]

Sex :

- ○ Women
- ○ Men
- ○ Unknown
- ○ Undetermined

Are you an Aboriginal person (First Nation, Métis, or Inuk (Inuit))?

- ☐ No, not an Aboriginal person
- ☐ Yes, First Nation
- ☐ Yes, Métis
- ☐ Yes, Inuk (Inuit)

What is your country of birth?

- ○ Canada
- ○ Other (please specify):

[ ]

Are you? (check all that apply):

- [ ] White
- [ ] South Asian - e.g., East Indian, Pakistani, Sri Lankan
- [ ] Chinese
- [ ] Black
- [ ] Filipino
- [ ] Latin American
- [ ] Arab
- [ ] Southeast Asian - e.g., Vietnamese, Cambodian, Malaysian, Laotian
- [ ] West Asian - e.g., Iranian, Afghan
- [ ] Korean
- [ ] Japanese
- [ ] Other :

(Please specify)

What employment status best represents your situation?

- ◯ Currently working full-time
- ◯ Currently working part-time
- ◯ Looking for work / Unemployed
- ◯ On sick leave
- ◯ On maternity leave
- ◯ Temporarily or permanently disabled due to your pain
- ◯ Disabled for reasons other than pain
- ◯ Student
- ◯ Temporarily laid off (planned unemployment)
- ◯ Retired
- ◯ Homemaker
- ◯ Unknown
- ◯ Other :
- ◯ (Please specify)

Are you currently involved in a legal dispute to obtain disability benefits?
For example, with your employer, an insurance company, from the Commission des normes, de l'équité, de la santé et de la sécurité du travail (CNESST), from the Société de l'Assurance automobile du Québec (SAAQ), or from the Indemnisation des victimes d'actes criminels (IVAC).

- ◯ Yes
- ◯ No
- ◯ Uncertain

Education level (highest level successfully completed) :

- ◯ Without a high school diploma
- ◯ High school (secondary school) diploma or equivalent
- ◯ Registered apprenticeship or other trades certificate or diploma (e.g., hairstyling, cooking, electrician, carpentry)
- ◯ College diploma or certificate from a CEGEP or other non-university institution (e.g., accounting technology, industrial engineering technology, legal assistant, pre-university DEC, etc.)
- ◯ Undergraduate university program (e.g., bachelor's degree, certificate)
- ◯ Graduate university program (e.g., master's degree, D.E.S.S., graduate microprogram)
- ◯ Doctorate (e.g., MD, PharmD, Ph.D., Psy.D., Ed.D.)

In which region of Quebec do you currently live?

- ◯ Abitibi-Témiscamingue
- ◯ Bas-Saint-Laurent
- ◯ Capitale Nationale (Quebec City and surrounding areas)
- ◯ Centre-du-Québec (Central Quebec)
- ◯ Chaudière-Appalaches
- ◯ Côte-Nord (North Shore)
- ◯ Estrie (Eastern Townships)
- ◯ Gaspésie-Îles-de-la-Madeleine (Gaspésie-Magdalen Islands)
- ◯ Lanaudière
- ◯ Laurentides
- ◯ Laval
- ◯ Mauricie
- ◯ Montérégie
- ◯ Montréal
- ◯ Nord-du-Québec
- ◯ Outaouais
- ◯ Saguenay-Lac-Saint-Jean
- ◯ Other (Please specify): ____________

Please indicate how much each of the following characteristics represents you:

	1 (Never or almost never true)	2	3	4	5	6	7 (always or almost always true)
Self-reliant	◯	◯	◯	◯	◯	◯	◯
Yielding	◯	◯	◯	◯	◯	◯	◯
Athletic	◯	◯	◯	◯	◯	◯	◯

	1 (jamais ou presque jamais vrai)	2	3	4	5	6	7 (presque toujours vrai)
Affectionate	○	○	○	○	○	○	○
Assertive	○	○	○	○	○	○	○
Forceful	○	○	○	○	○	○	○
Sympathetic	○	○	○	○	○	○	○
Has leadership abilities	○	○	○	○	○	○	○
Sensitive to the needs of others	○	○	○	○	○	○	○
Compassionate	○	○	○	○	○	○	○
Eager to soothe hurt feelings	○	○	○	○	○	○	○
Dominant	○	○	○	○	○	○	○
Warm	○	○	○	○	○	○	○
Tender	○	○	○	○	○	○	○
Acts as a leader	○	○	○	○	○	○	○
Competitive	○	○	○	○	○	○	○
Loves children	○	○	○	○	○	○	○
Gentle	○	○	○	○	○	○	○

UQAT
LABORATOIRE DE RECHERCHE
EN ÉPIDÉMIOLOGIE DE LA
DOULEUR CHRONIQUE

Better understanding chronic pain and its treatment

General health information

In general, would you say your health is:

- ◯ Excellent
- ◯ Very good
- ◯ Good
- ◯ Fair
- ◯ Poor

How many different medications are you currently using (whether they are prescription or over-the-counter; whether for pain or any other health condition)?

[ ]

Over the last 2 weeks, how often have you been bothered by the following problems?

	Not at all	Several days	More than half the days	Nearly every day
Feeling nervous, anxious or on edge	◯	◯	◯	◯
Not being able to stop or control worrying	◯	◯	◯	◯
Little interest or pleasure in doing things	◯	◯	◯	◯
Feeling down, depressed, or hopeless	◯	◯	◯	◯

The following questions are about activities you might do during a typical day. Does your current health limit you in these activities? If so, how much?

	Yes, limited a lot	Yes, limited a little	No, not limited at all
Moderate activities, such as moving a table, pushing a vacuum cleaner, bowling, or playing golf	◯	◯	◯
Climbing several flights of stairs	◯	◯	◯

In the past year...

	Never	Rarely	Sometimes	Often
Have you consumed alcohol or used drugs more than you meant to?	◯	◯	◯	◯
Have you ever felt that you ought to cut down on your drinking or drug use?	◯	◯	◯	◯

In the past year, have you used cannabis?

	Yes	No
For your pain	◯	◯
For other health issues	◯	◯
For recreational purposes (for pleasure)	◯	◯

How would you describe your smoking habits (cigarettes)?

◯ Never smoked

◯ Current smoker

◯ Used to smoke, but have now quit

Height :

In feet/inches (e.g., 5'6"): ____________________

OR

In centimeters (cm): ____________________

Weight:

In pounds (lbs): [ ]

OR

In kilograms (kg): [ ]

How did you hear about this survey?

- ◯ Facebook
- ◯ Twitter
- ◯ Instagram
- ◯ Email received from a friend, family member, coworker, or professor
- ◯ Email received from a patient association (e.g., Quebec Association for Chronic Pain, Fibromyalgia Association, Arthritis Society)
- ◯ Website of the Université du Québec en Abitibi-Témiscamingue (UQAT)
- ◯ Website of a radio station
- ◯ Heard on the radio
- ◯ Print newspaper
- ◯ Other (please specify):

[ ]

The questionnaire on pain and its management ends here

Would you like to receive summaries of the project results?

◯ Yes ◯ No

Would you be willing to be contacted again for future studies conducted by our research group?

◯ Yes ◯ No

If you answered yes to any of the previous three questions, please enter your email address in the box below:

[ ]

If you do not have an email address, you can still provide us with your postal address:

Name	
Adress	
Adress Line 2	
City / Town	
Province	
Postal Code	
Country	

Thank you very much for your participation!

We are very grateful that you agreed to help us with our research!

UQAT

LABORATOIRE DE RECHERCHE
EN ÉPIDÉMIOLOGIE DE LA
DOULEUR CHRONIQUE

SUPPLEMENTARY CONTENT 2. Autoclustering BIC values by number of clusters

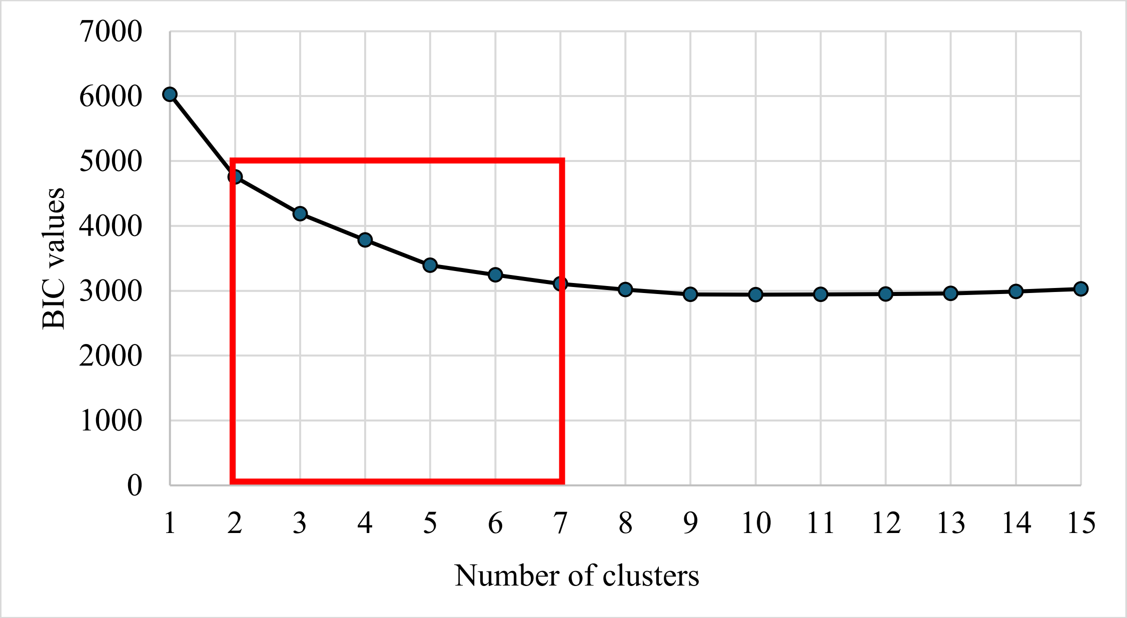

SUPPLEMENTARY CONTENT 3. Integral results of the multivariable model exploring associations between subgroups and frequent medical care utilization.

Variable	Odds ratio (OR) – Adjusted β	p-value	95% CI
Subgroups (vs subgroup 1: men)			
Subgroup 2 – Women living in remote region	1.25	0.72	0.37-4.30
Subgroup 3 – Employed more educated women	2.50	0.11	0.80-7.75
Subgroup 4 – Unemployed more educated women	**3.93**	**0.01**	**1.44-10.80**
Subgroup 5 – Less educated women	2.98	0.06	0.94-9.47
Pain duration in year (continuous)	0.99	0.55	0.97-1.02
Pain intensity in the last 7 days (vs. mild; score 1-4)			
Moderate (score 5-7)	1.04	0.92	0.52-2.06
Severe (score 8-10)	1.06	0.90	0.41-2.73
Multisite pain (≥2 sites) (yes vs. no)	0.99	0.99	0.39-2.50
Generalized pain (yes vs. no)	0.98	0.94	0.54-1.76
Pain frequency (occasionally vs. continuously)	1.51	0.40	0.58-3.91
Agreeing with the statement ‘’ I feel that my pain is terrible and it’s never going to get any better’’ (yes vs. no)	1.38	0.31	0.73-2.6
Evidence of neuropathic pain according to the DN4 scale (yes vs. no)	1.35	0.31	0.76-2.38
Pain interference according to the BPI score (continuous)	**1.38**	**<0.001**	**1.15-1.67**
Pharmacological pain treatments use (yes vs. no)	0.88	0.67	0.49-1.57

Excessive polypharmacy (≥10 medications) (yes vs. no)	0.63	0.09	0.36-1.08
Side effects associated with medications (yes vs. no)	1.12	0.74	0.57-2.19
Use of cannabis for pain management (yes vs. no)	0.89	0.685	0.498-1.58
Physical and/or psychological pain treatments use (yes vs. no)	0.65	0.32	0.28-1.52
Access to a trusted health care professional for pain management (yes vs. no)	**2.94**	**0.02**	**1.18-7.30**
Drug and alcohol use (Have you consumed alcohol or used drugs more than you meant to vs. never)			
Rarely	1.08	0.82	0.55-2.12
Sometimes	0.84	0.70	0.36-2.00
Often	1.22	0.69	0.45-3.32
Smoking habits (vs no smokers)			
Smokers	0.84	0.67	0.38-1.87
I smoked in the past, but not anymore	0.61	0.10	0.33-1.10
Anxiety-Depression symptoms – PHQ Score (vs. none; score 0-2)			
Mild (score 3-5)	0.59	0.18	0.28-1.27
Moderate (score 6-8)	0.71	0.40	0.31-1.59
Severe (score 9-12)	0.73	0.48	0.30-1.75
Having private prescription drug insurance (yes vs. no)	0.58	0.06	0.32-1.03

Comorbidity score (Charlson & Elixhauser index) (continuous)	0.91	0.68	0.59-1.41

Table footnotes: p-values < 0.05 are reported in **bold**, 95% CI: 95% confidence interval and OR: odds ratio. The multivariable analysis was adjusted for the following covariables: pain duration, pain intensity in the last 7 days, multisite pain, generalized pain, pain frequency, agreeing with the statement ''I feel that my pain is terrible and it's never going the get any better'', evidence of neuropathic pain according to the DN4 scale, pain interference according to the BPI score, pharmacological pain treatments use, excessive polypharmacy (≥10 medications), side effects associated with medication, use of cannabis for pain management, physical/psychological pain treatments use, access to a trusted health care professional for pain management, feeling the need to reduce alcohol or drug consumption, smoking habits, psychological distress according to the PHQ scale, having private prescription drug insurance, and comorbidity score (Charlson & Elixhauser index). In total, 717 participants (80.1%) were included in the final model (178 missing data; 19.9% were excluded).
